# Supplementary material for: Structural dynamics in the evolution of SARS-CoV-2 spike glycoprotein
Source: Nat Commun. 2023 Mar 14;14:1421. doi: 10.1038/s41467-023-36745-0 (PMC10013288; doi:10.1038/s41467-023-36745-0)
Supplement: Supplementary file 1 — Supplementary Information [file 41467_2023_36745_MOESM1_ESM.pdf]

Structural dynamics in the evolution of SARS-CoV-2 spike glycoprotein

Supplementary information

Valeria Calvaresi[1]*, Antoni G. Wrobel[2]*, Joanna Toporowska[1], Dietmar Hammerschmid[1], Katie J. Doores[3], Richard T. Bradshaw[1], Ricardo B. Parsons[1], Donald J. Benton[2], Chloë Roustan[4], Eamonn Reading[1], Michael H. Malim[3], Steven J. Gamblin[2], Argyris Politis[1,5,6]*

*Correspondence to:

valeria.clavaresi@kcl.ac.uk antoni.wrobel@crick.ac.uk argyris.politis@manchester.ac.uk

[1] Department of Chemistry, King's College London, SE1 1DB, London, UK

[2] Structural Biology of Disease Processes Laboratory, The Francis Crick Institute, NW1 1AT, London, UK

[3] Department of Infectious Diseases, School of Immunology and Microbial Sciences, King's College London, SE1 9RT, London, UK

[4] Structural Biology Science Technology Platform, The Francis Crick Institute, NW1 1AT London, UK

[5] Faculty of Biology, Medicine and Health, School of Biological Sciences, The University of Manchester, M13 9PT, Manchester, UK

[6] Manchester Institute of Biotechnology, The University of Manchester, M1 7DN, Manchester, UK

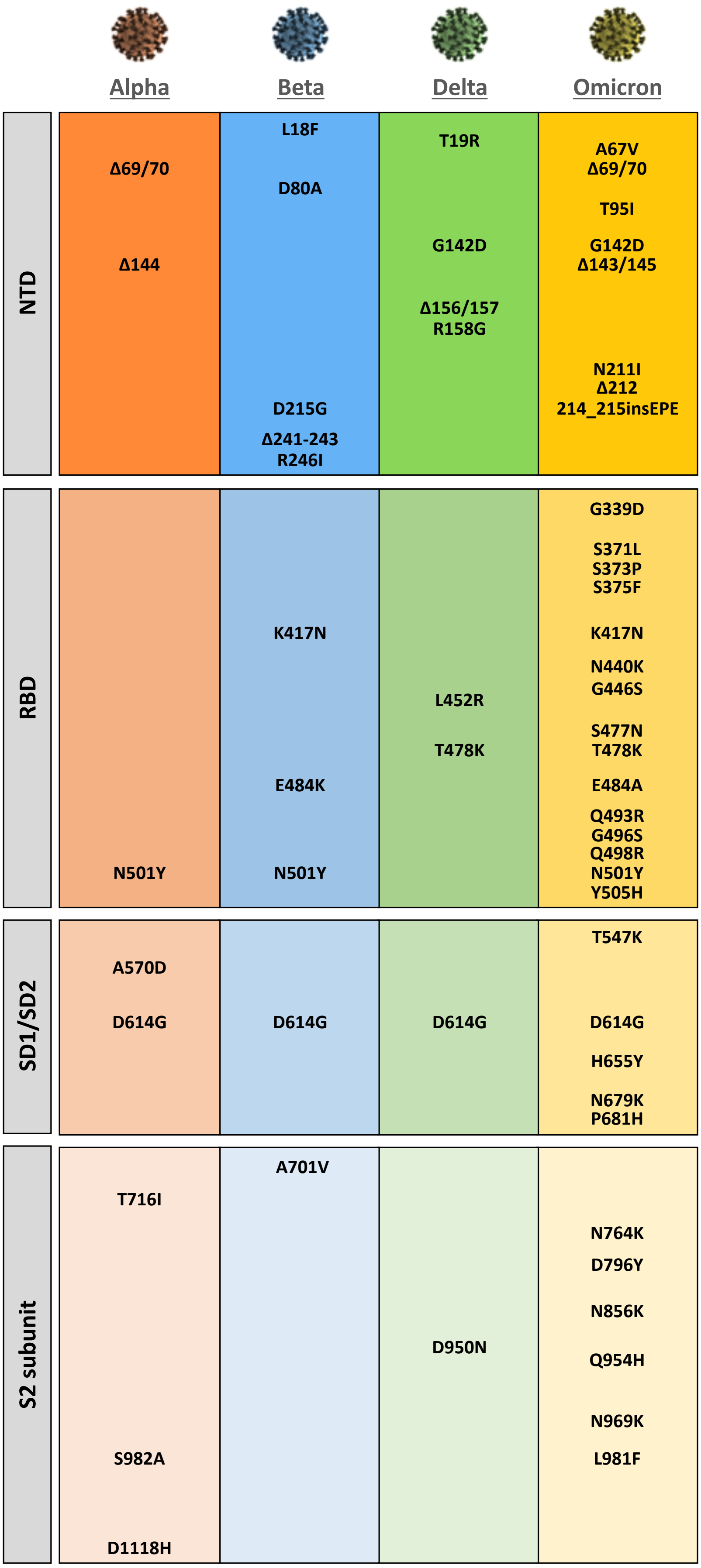

Supplementary Fig. 1. Mutations in spike genome of SARS-CoV-2 variants analysed.

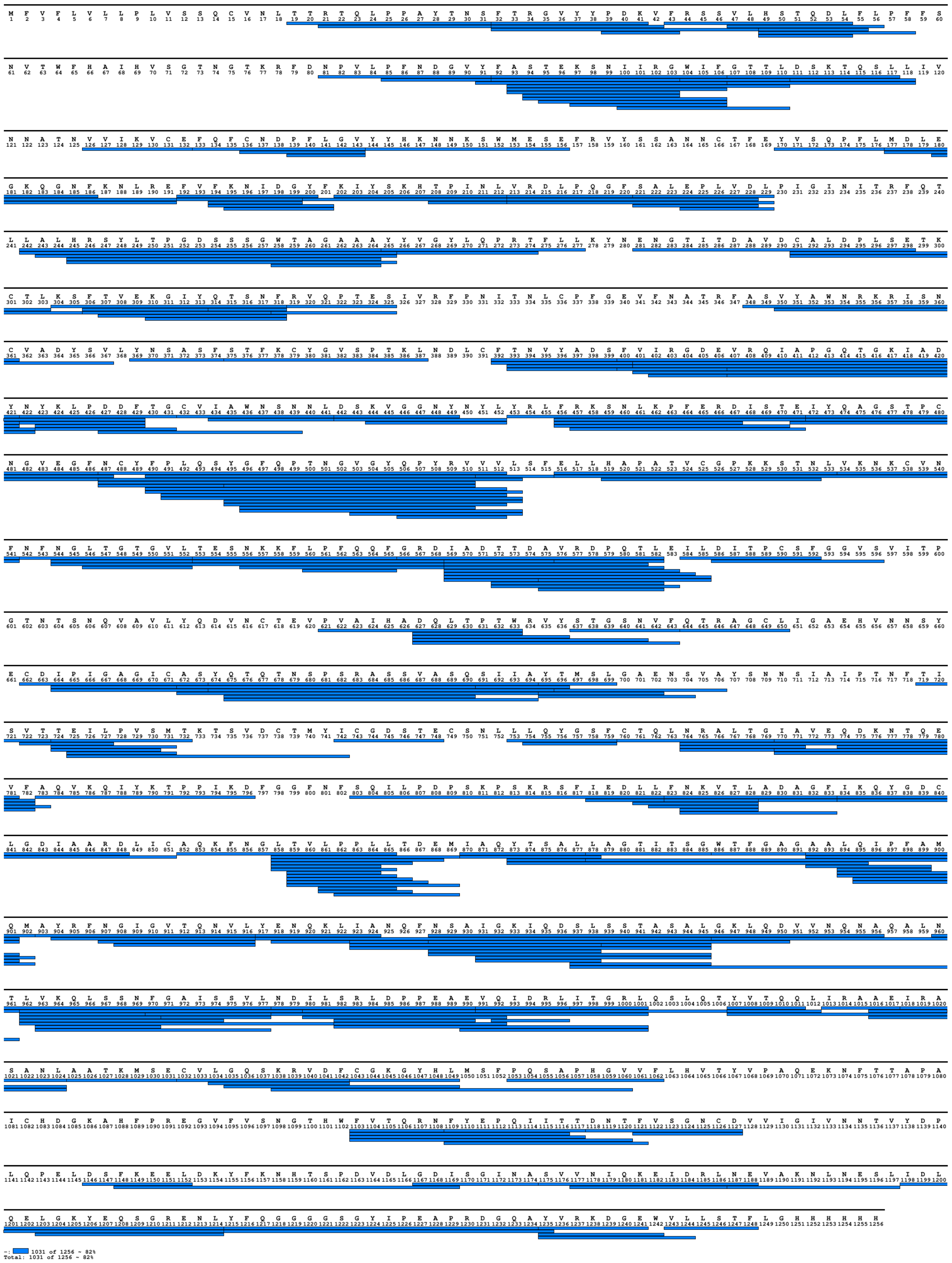

Supplementary Fig. 2. Coverage map of peptides whose HDX was followed for comparing Wuhan and G614 spikes. The signal peptide (residues 1-13) is indicated but not present in the protein analysed. The figure was generated with MS Tools[1].

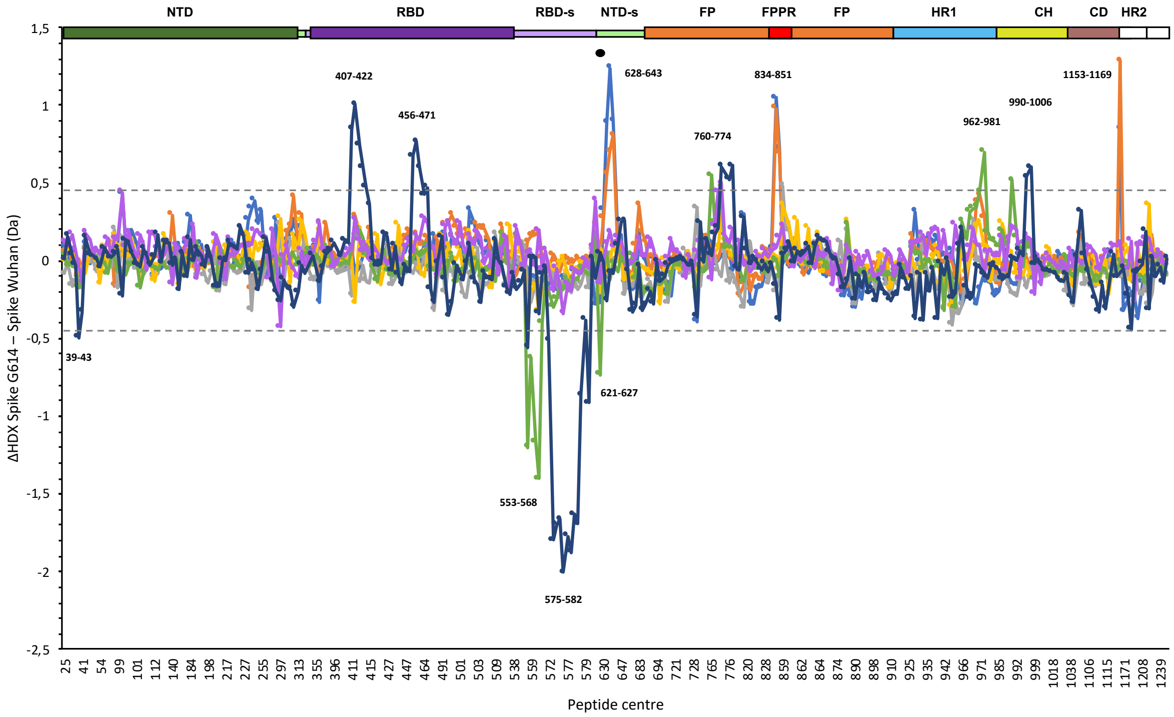

Supplementary Fig. 3. Difference plot illustrating the difference in HDX between Wuhan and G614 spikes (light blue line: 4 s on ice, orange line: 20 s on ice, grey line: 15 s at 23 °C, yellow line: 1 min at 23 °C, green line: 10 min at 23 °C, violet line: 100 min at 23 °C, dark blue line: 360 min at 28 °C). Residues comprising a region with significant differences in HDX are indicated. The peptides are arranged according to their peptide centre residue. A dotted grey line indicates the 98% CI as a threshold for significance. The top bar shows spike subdomains. The black dot indicates the position of D614G substitution. Source data are provided as a Source Data file.

a

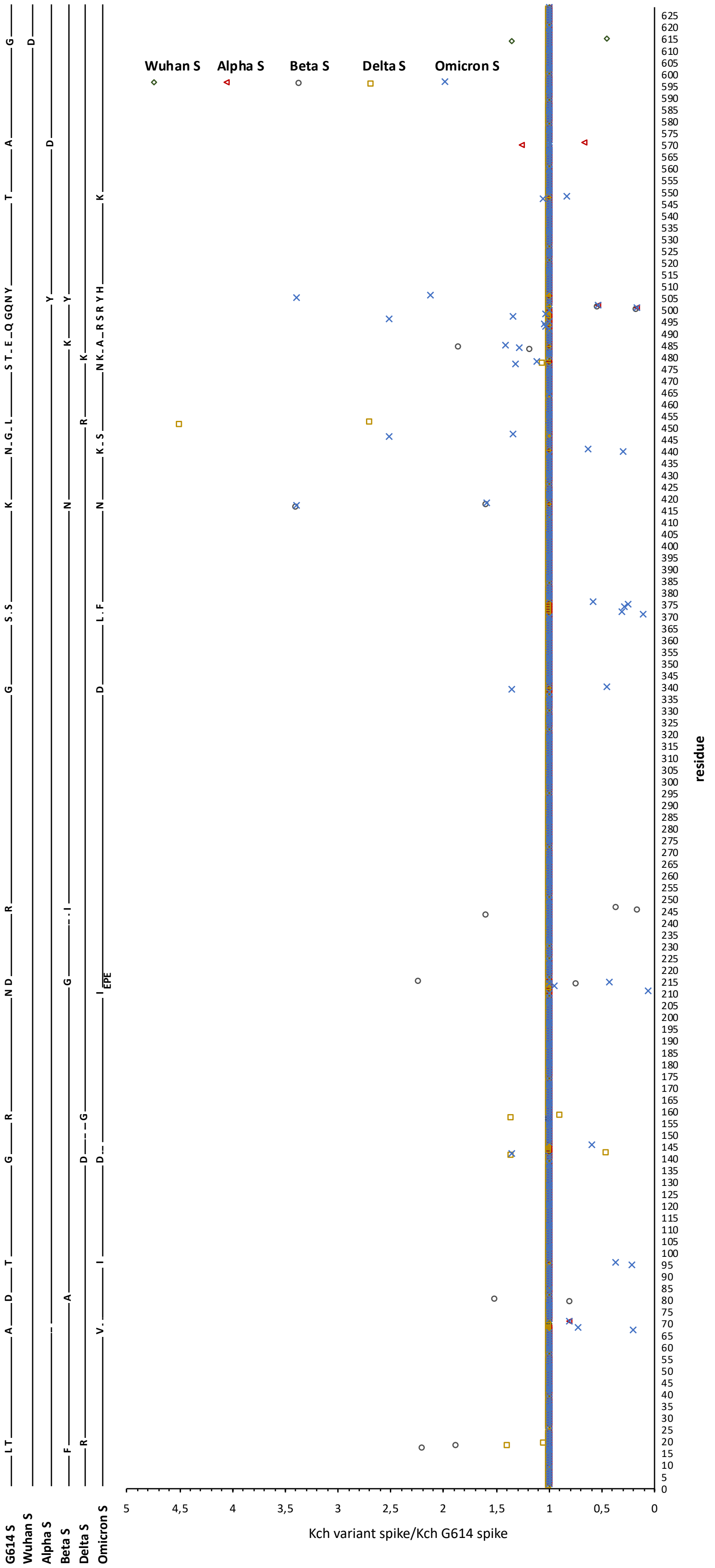

Supplementary Fig. 4. Influence of amino acid changes on the k_{ch} of residues of spike variants. The ratios between the k_{ch} of spike variants and Wuhan spike and the k_{ch} of G614 spike residues is plotted from residue 1 to 628 (a) and from residue 629 to 1256 (b). Values are extracted from Supplementary Data 1. Amino acid changes are illustrated on the left of the graphs.

b

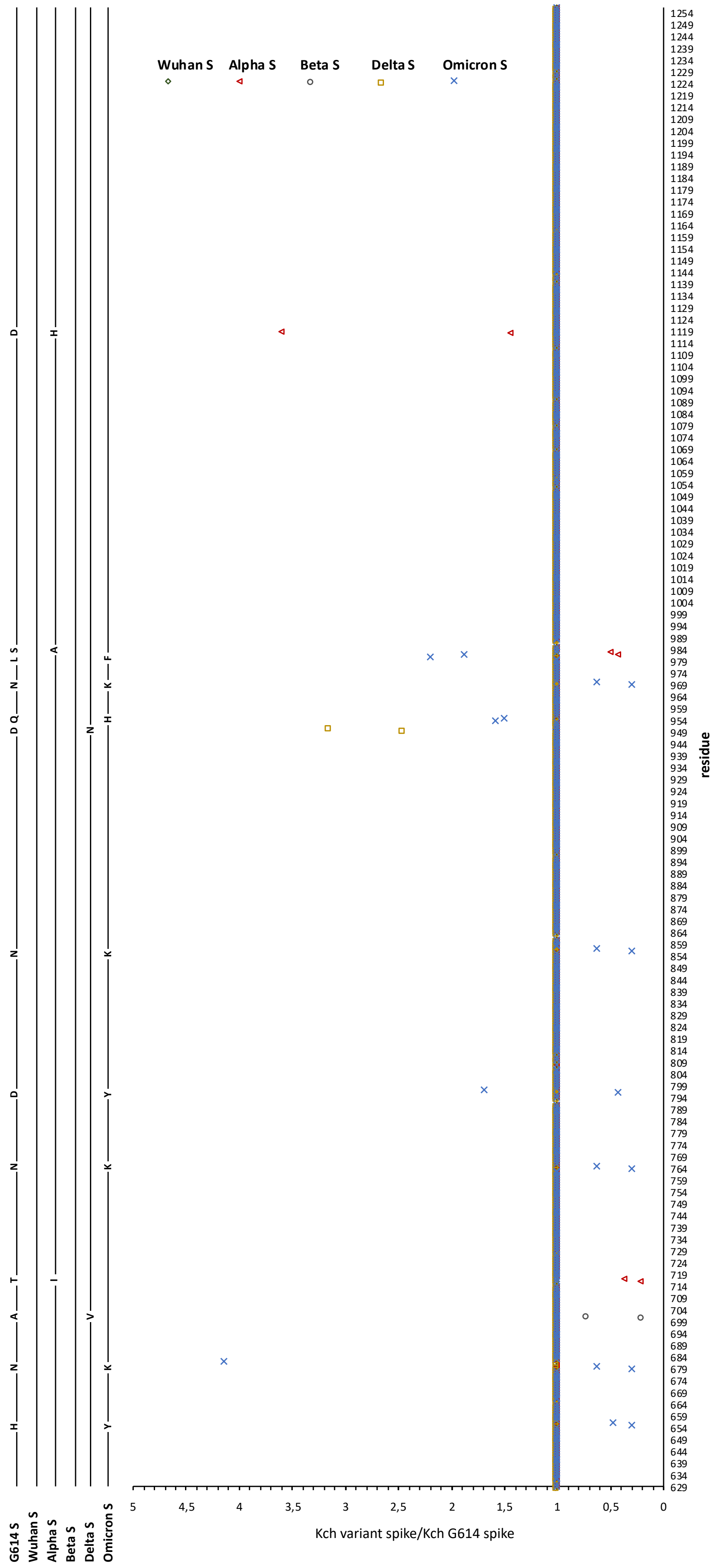

Wuhan S
Alpha S
Beta S
Delta S
Omicron S
G614 S
Wuhan S
Alpha S
Beta S
Delta S
Omicron S
H N A T N D N D Q N L S D
I A H
V N
Y K K Y K H K F
5 4,5 4 3,5 3 2,5 2 1,5 1 0,5 0
Kch variant spike/Kch G614 spike
residue
1254 1249 1244 1239 1234 1229 1224 1219 1214 1209 1204 1199 1194 1189 1184 1179 1174 1169 1164 1159 1154 1149 1144 1139 1134 1129 1124 1119 1114 1109 1104 1099 1094 1089 1084 1079 1074 1069 1064 1059 1054 1049 1044 1039 1034 1029 1024 1019 1014 1009 1004 999 994 989 984 979 974 969 964 959 954 949 944 939 934 929 924 919 914 909 904 899 894 889 884 879 874 869 864 859 854 849 844 839 834 829 824 819 814 809 804 799 794 789 784 779 774 769 764 759 754 749 744 739 734 729 724 719 714 709 704 699 694 689 684 679 674 669 664 659 654 649 644 639 634 629

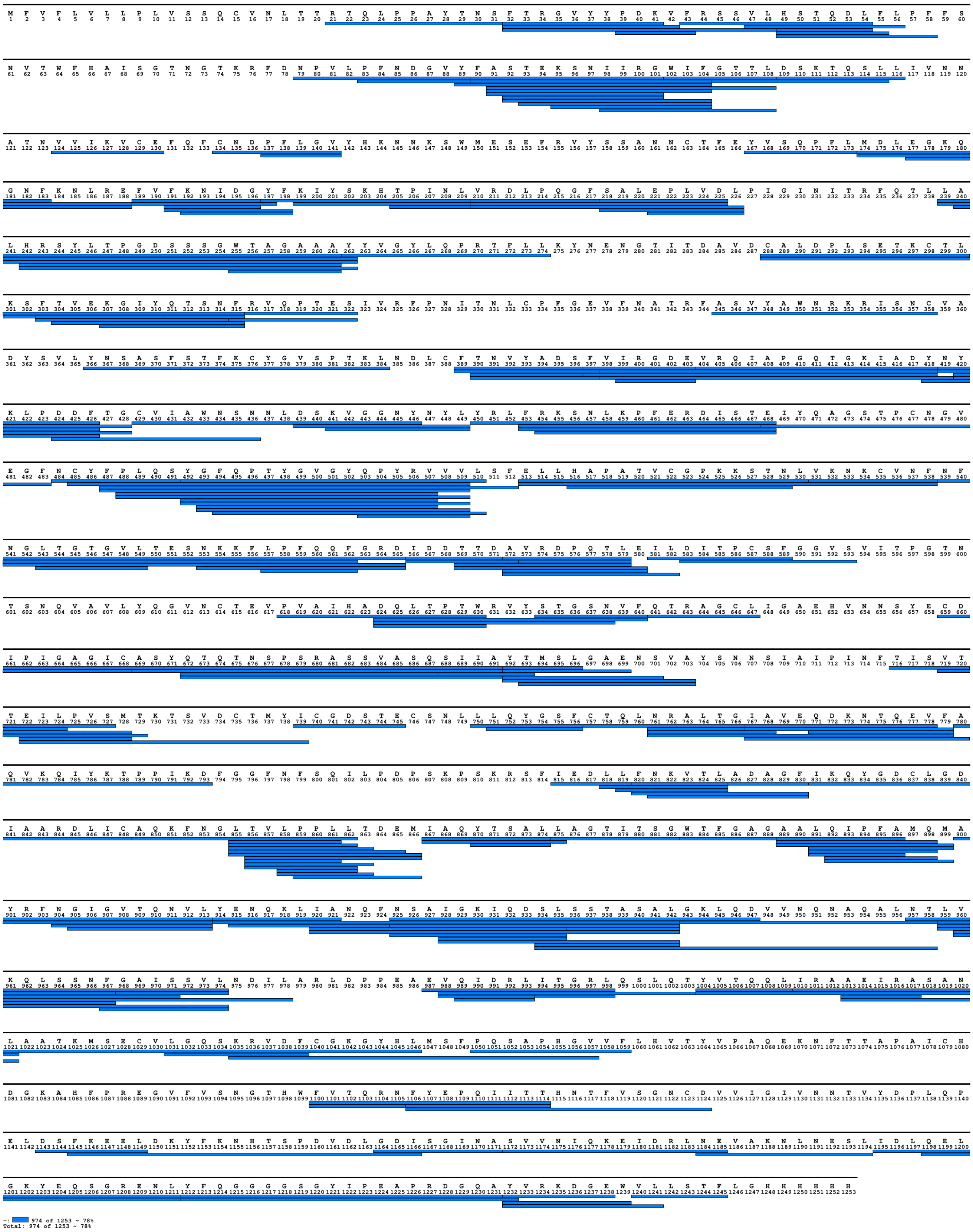

Supplementary Fig. 5. Coverage map of peptides whose HDX was followed in alpha spike (apo state). The signal peptide (residues 1-13) is indicated but not present in the protein analysed. The figure was generated with MS Tools.

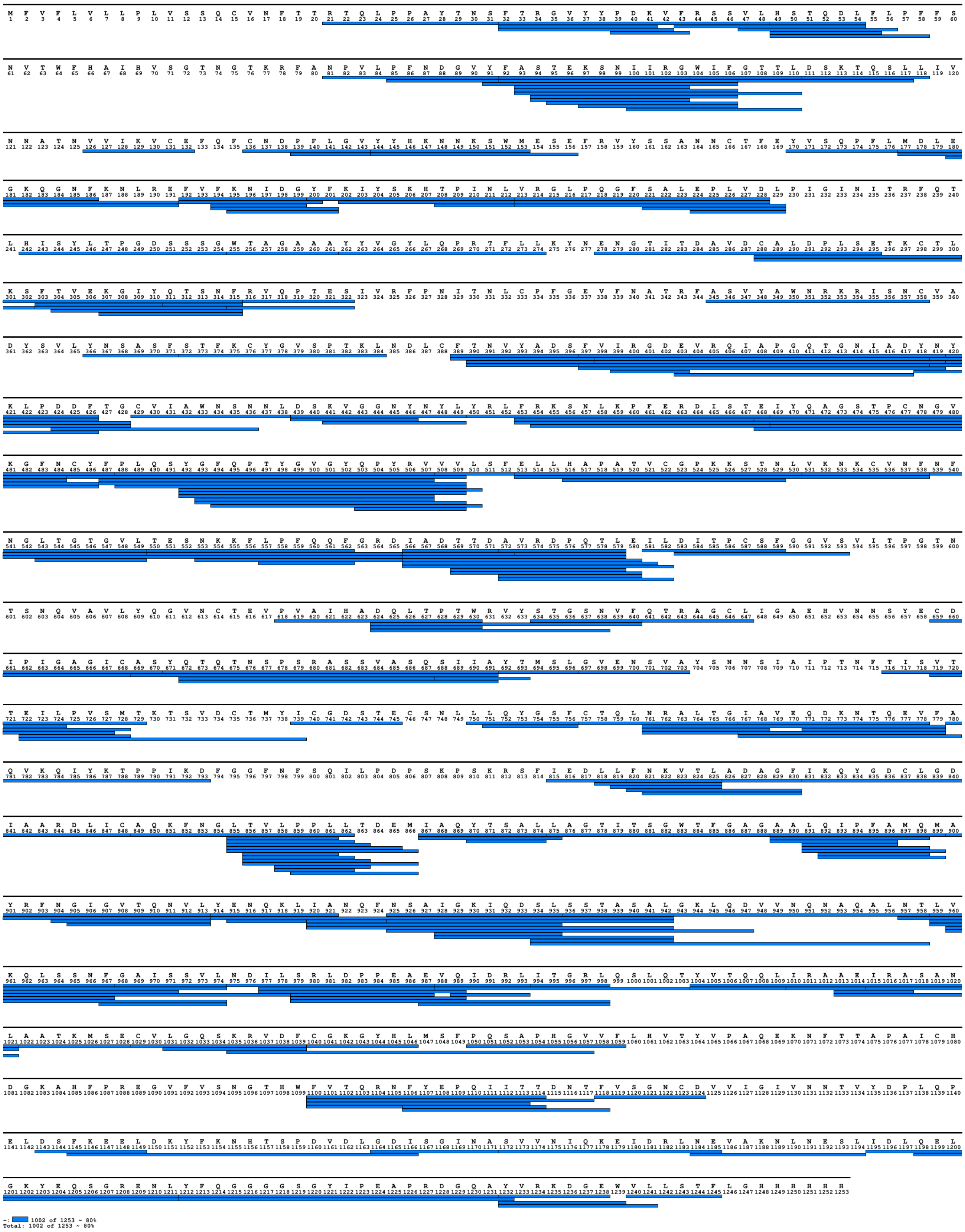

Supplementary Fig. 6. Coverage map of peptides whose HDX was followed in beta spike (apo state). The signal peptide (residues 1-13) is indicated but not present in the protein analysed. The figure was generated with MS Tools.

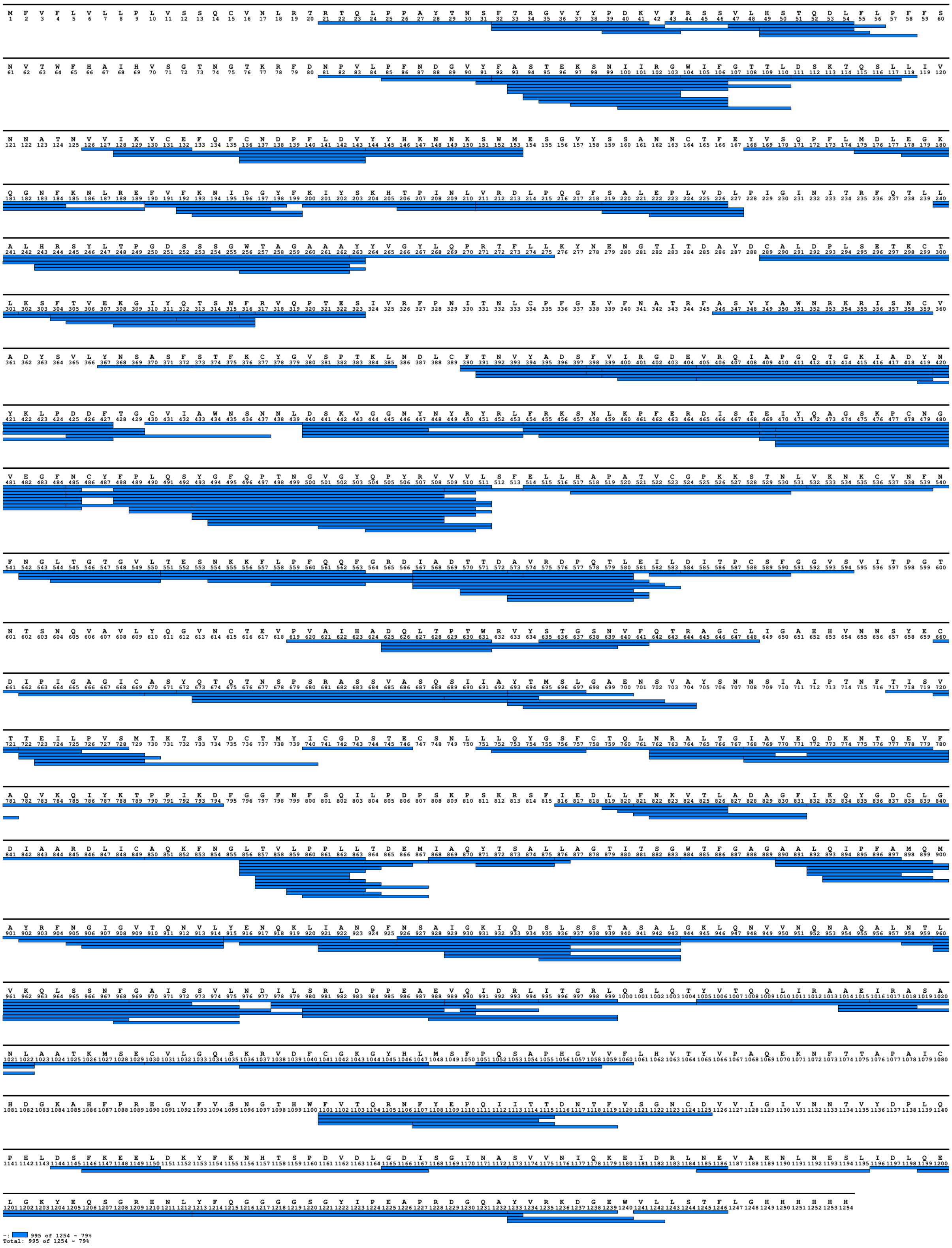

Supplementary Fig. 7. Coverage map of peptides whose HDX was followed in delta spike (apo state). The signal peptide (residues 1-13) is indicated but not present in the protein analysed. The figure was generated with MS Tools.

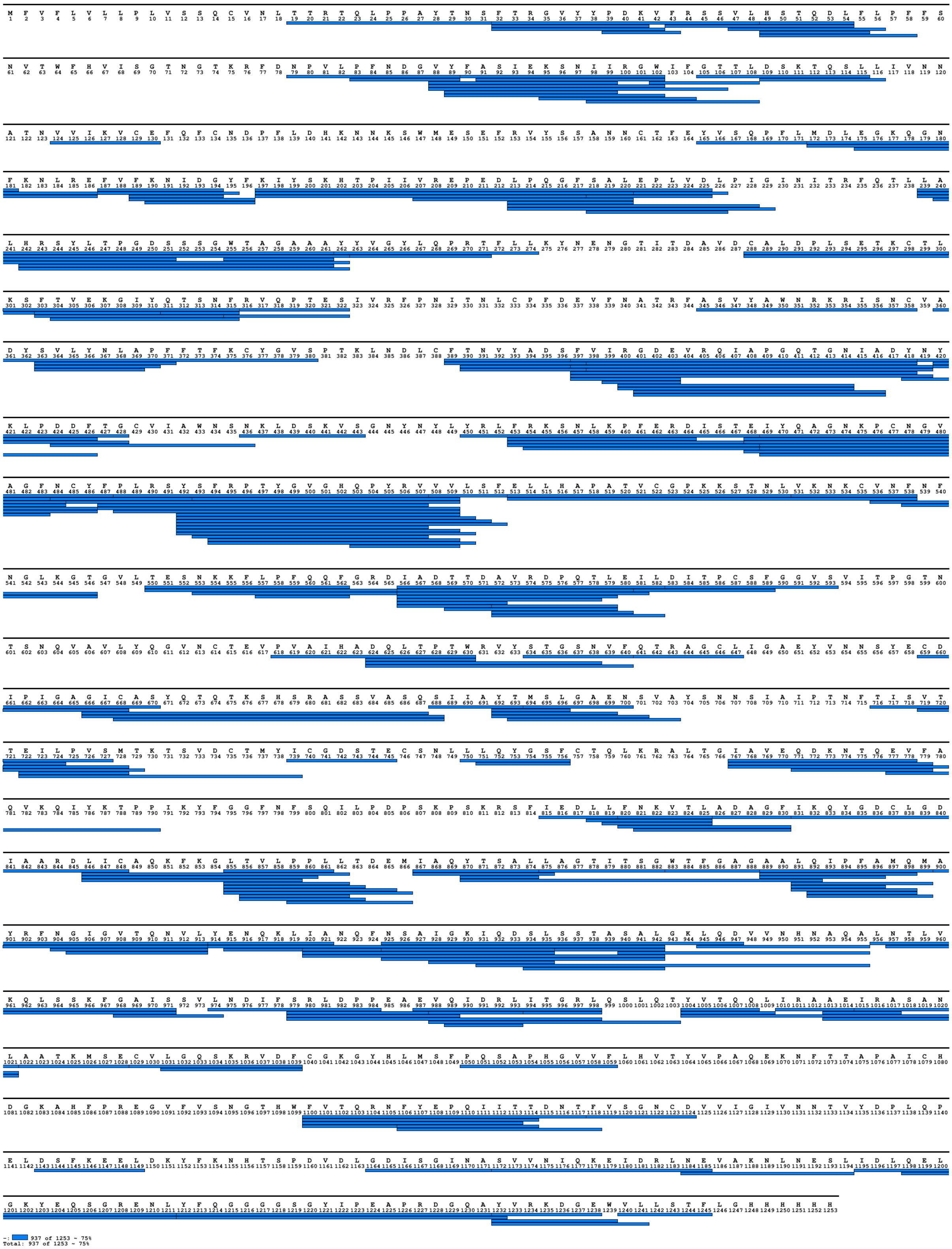

Supplementary Fig. 8. Coverage map of peptides whose HDX was followed in omicron spike (apo state). The signal peptide (residues 1-13) is indicated but not present in the protein analysed. The figure was generated with MS Tools.

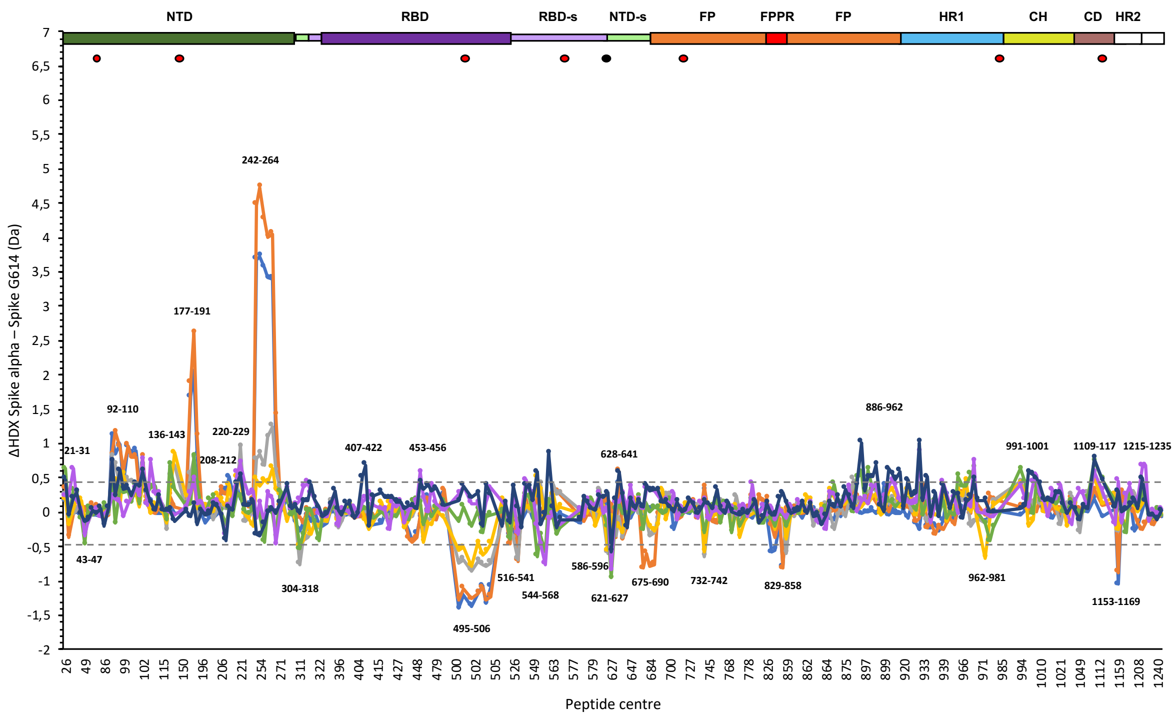

Supplementary Fig. 9. Difference plot illustrating the difference in HDX between alpha and G614 spikes (light blue line: 4 s on ice, orange line: 20 s on ice, grey line: 15 s at 23 °C, yellow line: 1 min at 23 °C, green line: 10 min at 23 °C, violet line: 100 min at 23 °C, dark blue line: 360 min at 28 °C). Residues comprising a region with significant differences in HDX are indicated according to residue numbering of G614 spike. The peptides are arranged according to their peptide centre residue. A dotted grey line indicates the 98% CI as a threshold for significance. The top bar shows spike subdomains. The black dot indicates the position of D614G substitution, whereas the red dots indicate the position of the other residue changes. Source data are provided as a Source Data file.

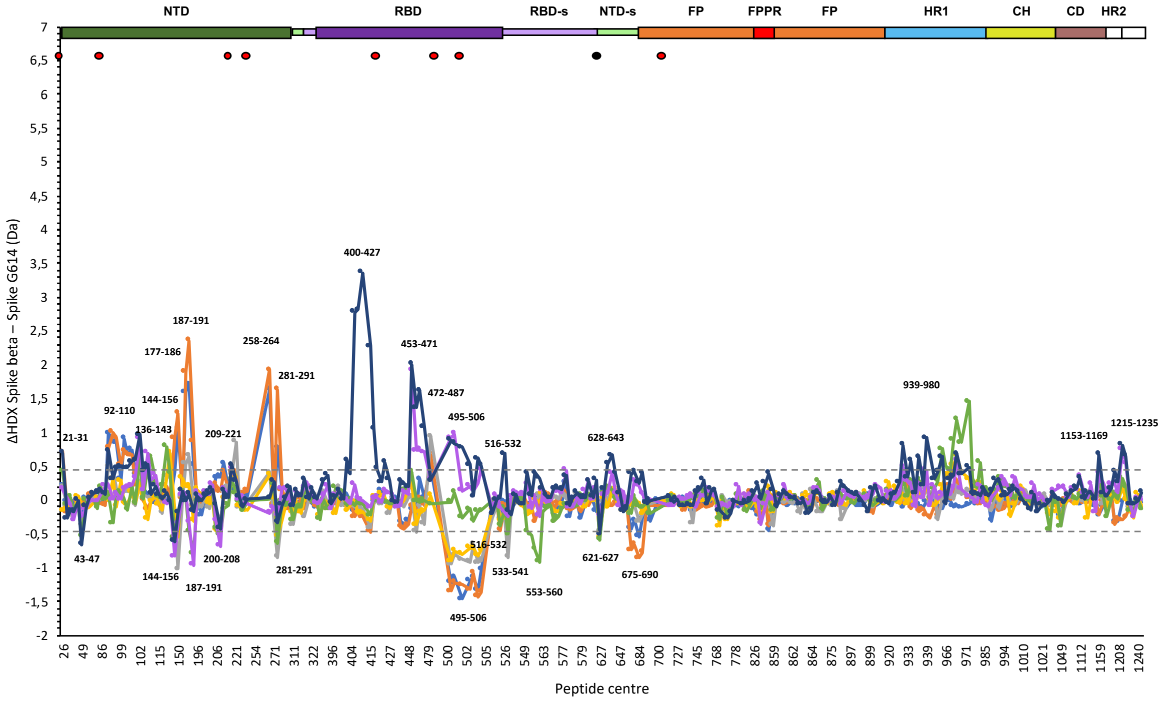

Supplementary Fig. 10. Difference plot illustrating the difference in HDX between beta and G614 spikes (light blue line: 4 s on ice, orange line: 20 s on ice, grey line: 15 s at 23 °C, yellow line: 1 min at 23 °C, green line: 10 min at 23 °C, violet line: 100 min at 23 °C, dark blue line: 360 min at 28 °C). Residues comprising a region with significant differences in HDX are indicated according to residue numbering of G614 spike. The peptides are arranged according to their peptide centre residue. A dotted grey line indicates the 98% CI as a threshold for significance. The top bar shows spike subdomains. The black dot indicates the position of D614G substitution, whereas the red dots indicate the position of the other residue changes. Source data are provided as a Source Data file.

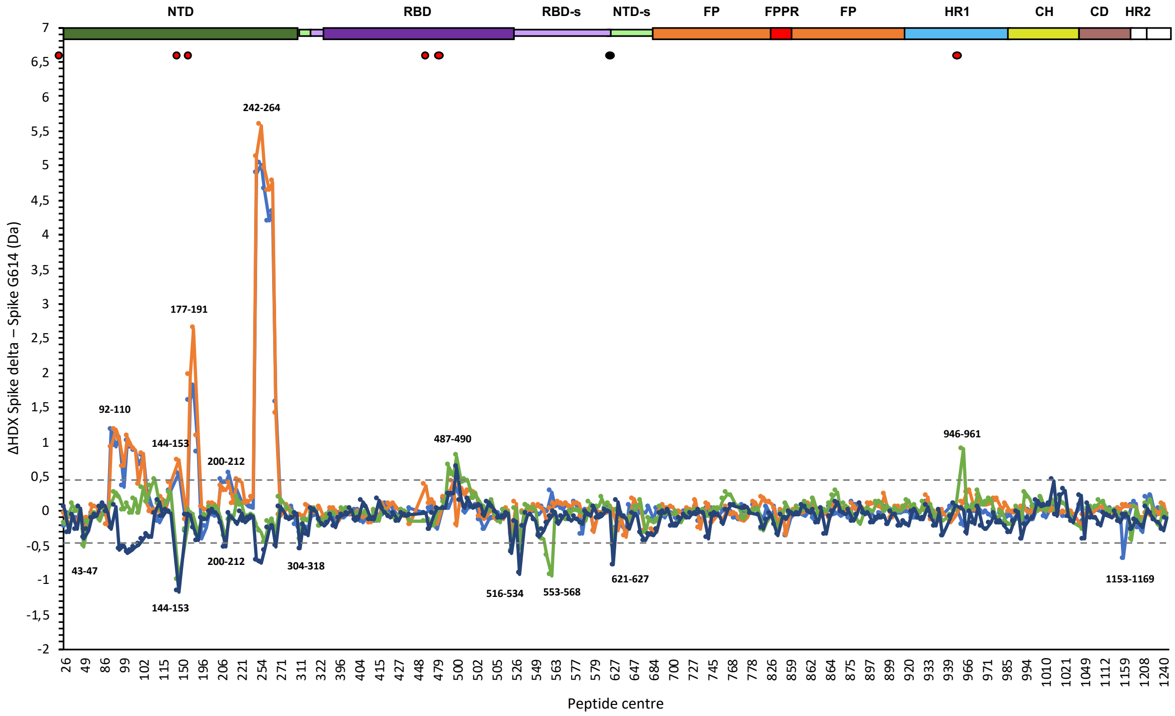

Supplementary Fig. 11. Difference plot illustrating the difference in HDX between delta and G614 spikes (light blue line: 4 s on ice, orange line: 20 s on ice, green line: 10 min at 23 °C, dark blue line: 360 min at 28 °C). Residues comprising a region with significant differences in HDX are indicated according to residue numbering of G614 spike. The peptides are arranged according to their peptide centre residue. A dotted grey line indicates the 98% CI as a threshold for significance. The top bar shows spike subdomains. The black dot indicates the position of D614G substitution, whereas the red dots indicate the position of the other residue changes. Source data are provided as a Source Data file.

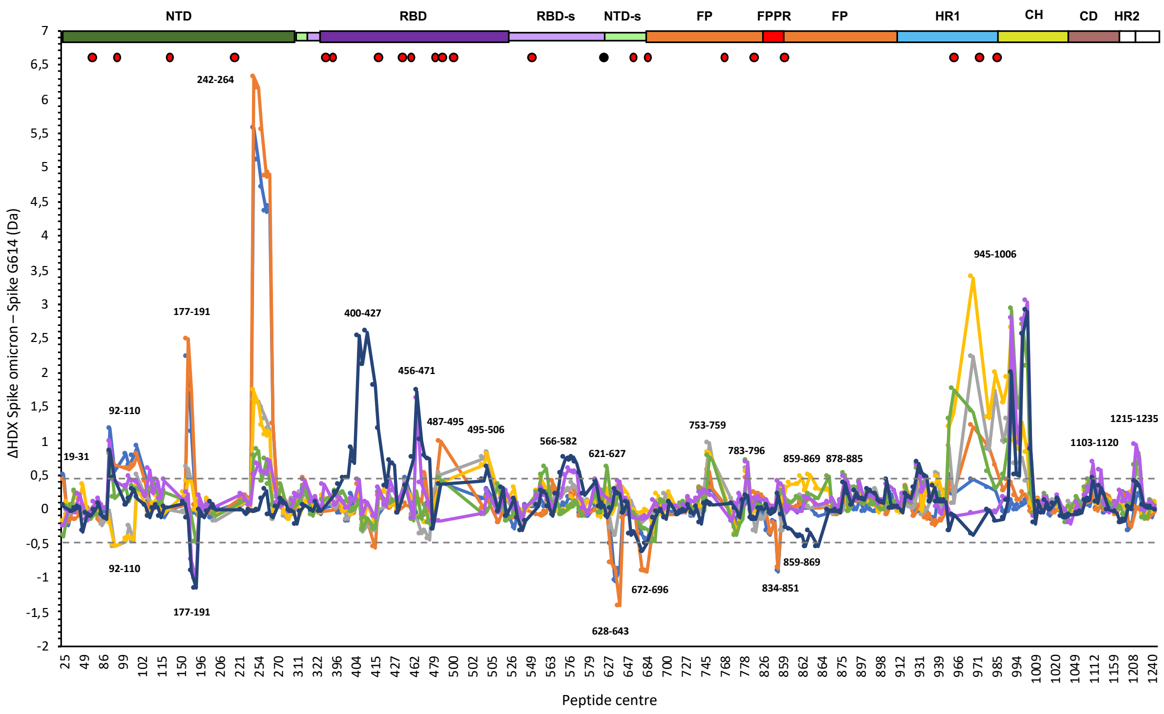

Supplementary Fig. 12. Difference plot illustrating the difference in HDX between omicron and G614 spikes (light blue line: 4 s on ice, orange line: 20 s on ice, grey line: 15 s at 23 °C, yellow line: 1 min at 23 °C, green line: 10 min at 23 °C, violet line: 100 min at 23 °C, dark blue line: 360 min at 28 °C). Residues comprising a region with significant differences in HDX are indicated according to residue numbering of G614 spike. The peptides are arranged according to their peptide centre residue. A dotted grey line indicates the 98% CI as a threshold for significance. The top bar shows spike subdomains. The black dot indicates the position of D614G substitution, whereas the red dots indicate the position of the other residue changes. Source data are provided as a Source Data file.

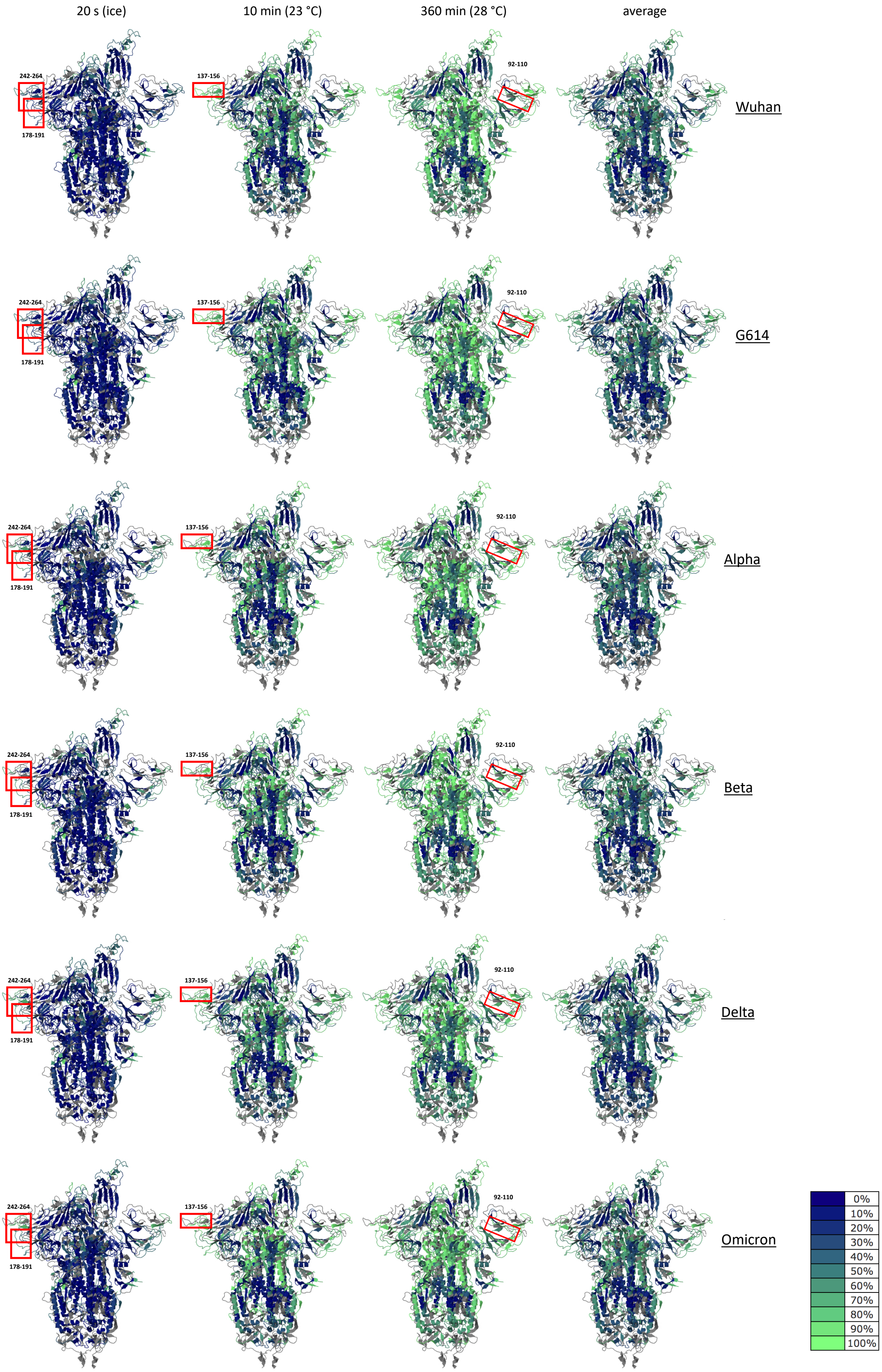

20 s (ice)
10 min (23 °C)
360 min (28 °C)
average
Wuhan
G614
Alpha
Beta
Delta
Omicron
242-264
178-191
137-156
92-110
0%
10%
20%
30%
40%
50%
60%
70%
80%
90%
100%

Supplementary Fig. 13. Conformational dynamics of Wuhan, G614, alpha, beta, delta and omicron spikes. Selected peptide segments are coloured on the structure of G614 spike with one protomer erected (PDB: 7bnn) in blue-to-green scale according to the percentage of deuterium uptake at selected time points (and an average of them) relative to the uptake of the maximally labelled control. Red squares frame protein regions displaying common effects in spike variants (in the protomer where they are better visible and at the time point when they more clearly manifest). Figures were generated with MS Tools and Jmol.

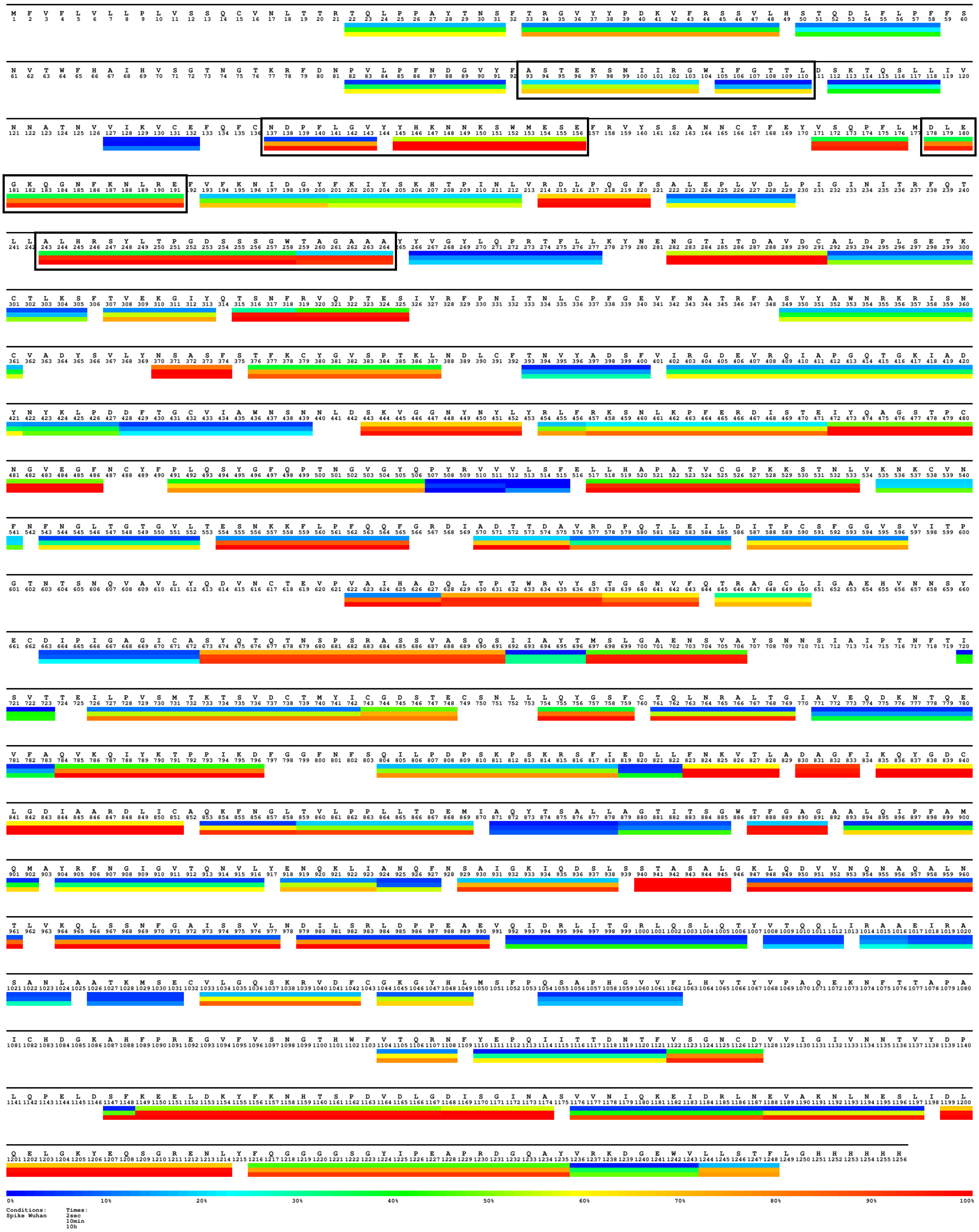

Supplementary Fig. 14. Heatmap of Wuhan spike for time points 20 s on ice (~2 s at 23 °C), 10 min at 23 °C and 360 min at 28 °C (~10 h at 23 °C). Selected peptide segments are coloured according to the percentage of deuterium uptake relative to the maximally labelled control. Peptide segments manifesting HDX in the NTD of spike variants are framed in black. The figure was generated with MS Tools.

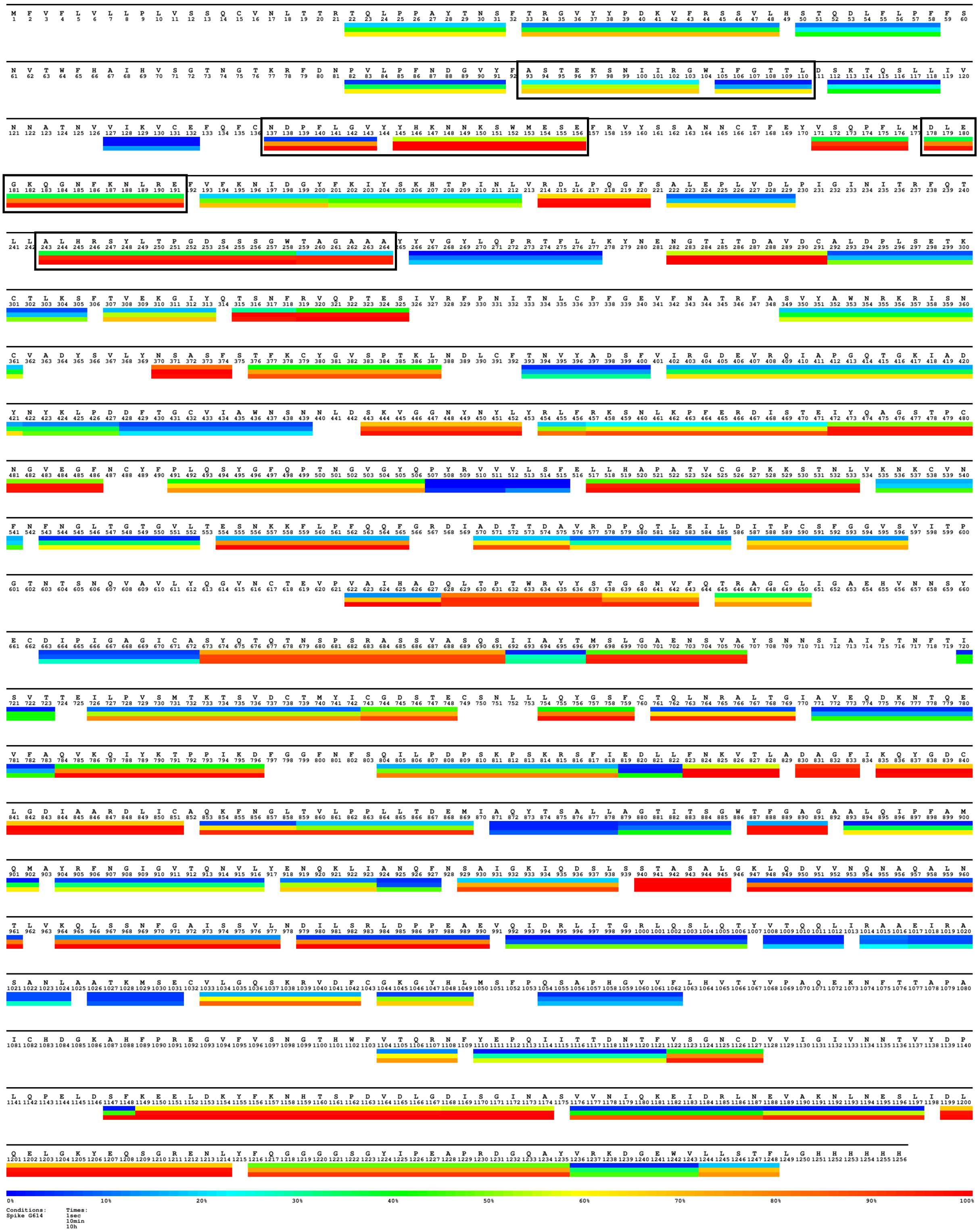

Supplementary Fig. 15. Heatmap of G614 spike for time points 20 s on ice (~2 s at 23 °C), 10 min at 23 °C and 360 min at 28 °C (~10 h at 23 °C). Peptide segments are coloured according to the percentage of deuterium uptake relative to the maximally labelled control. Selected peptide segments manifesting increased HDX in the NTD of spike variants are framed in black. The figure was generated with MS Tools.

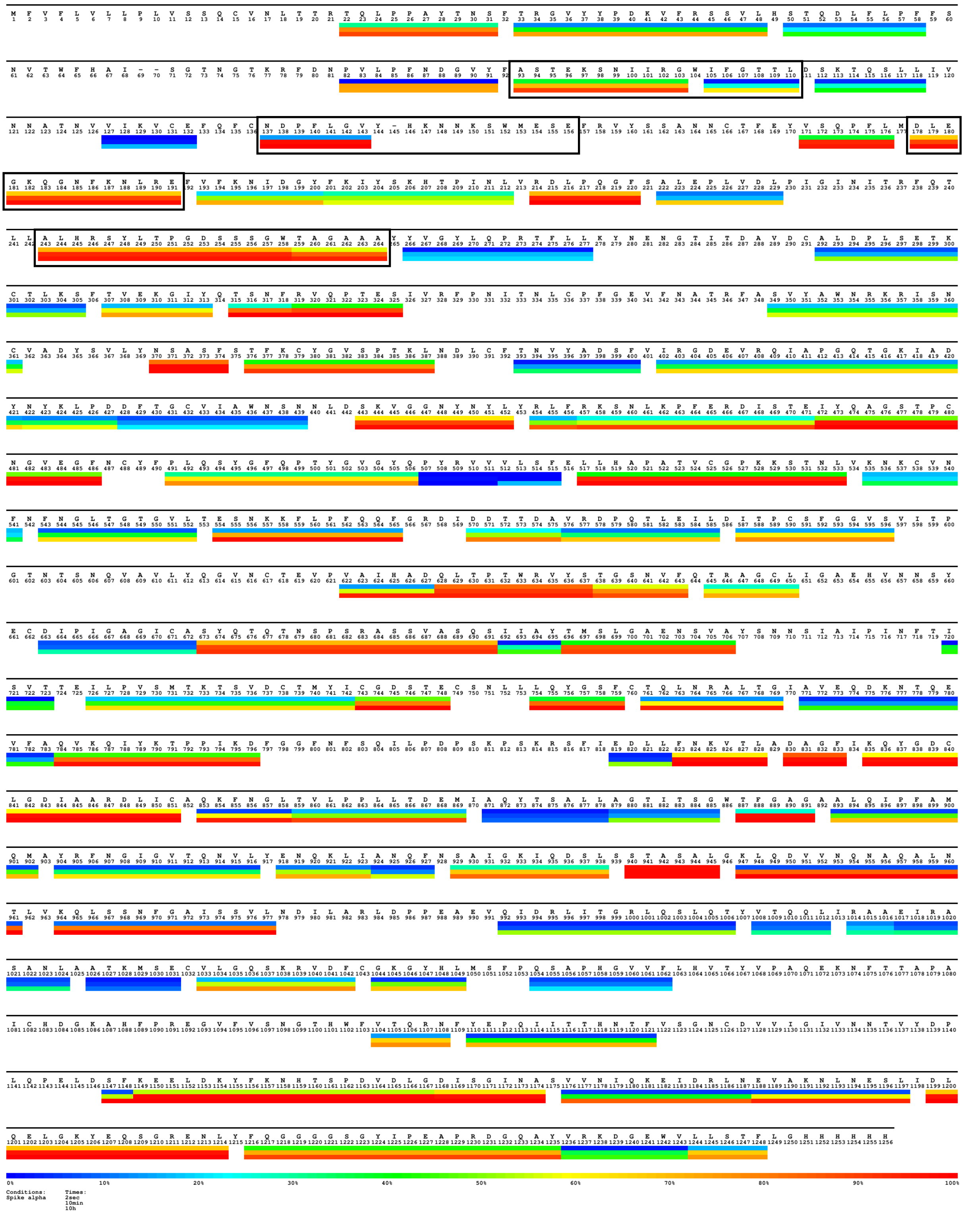

Supplementary Fig. 16. Heatmap of alpha spike for time points 20 s on ice (~2 s at 23 °C), 10 min at 23 °C and 360 min at 28 °C (~10 h at 23 °C). Selected peptide segments are coloured according to the % of uptake relative to the maximally labelled control. Sequence was adapted to the residue numbering of G614 spike. Peptide segments manifesting increased HDX in the NTD of spike variants are framed in black. The figure was generated with MS Tools.

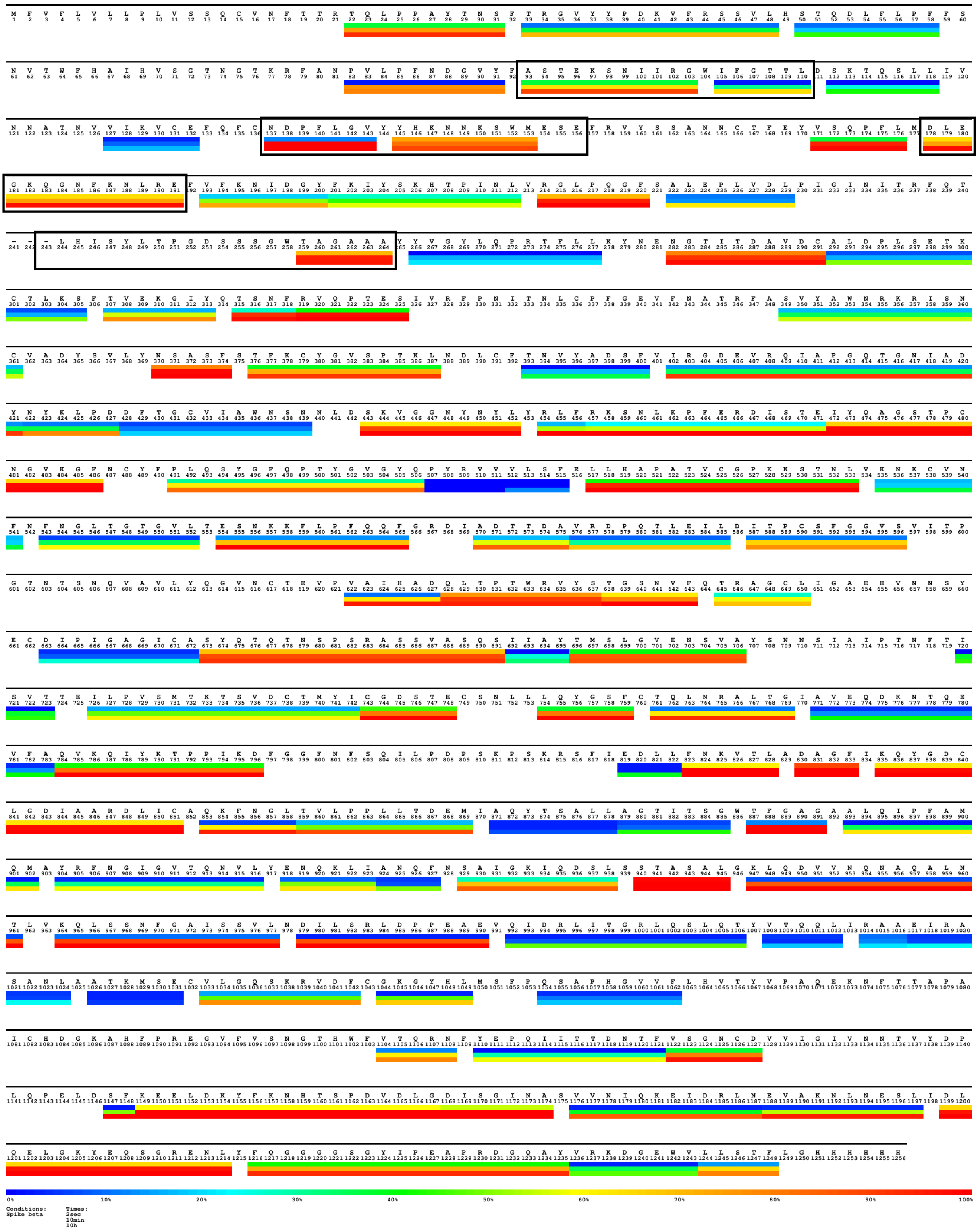

Supplementary Fig. 17. Heatmap of beta spike for time points 20 s on ice (~2 s at 23 °C), 10 min at 23 °C and 360 min at 28 °C (~10 h at 23 °C). Selected peptide segments are coloured according to the % of uptake relative to the maximally labelled control. Sequence was adapted to the residue numbering of G614 spike. Peptide segments manifesting increased HDX in the NTD of spike variants are framed in black. The figure was generated with MS Tools.

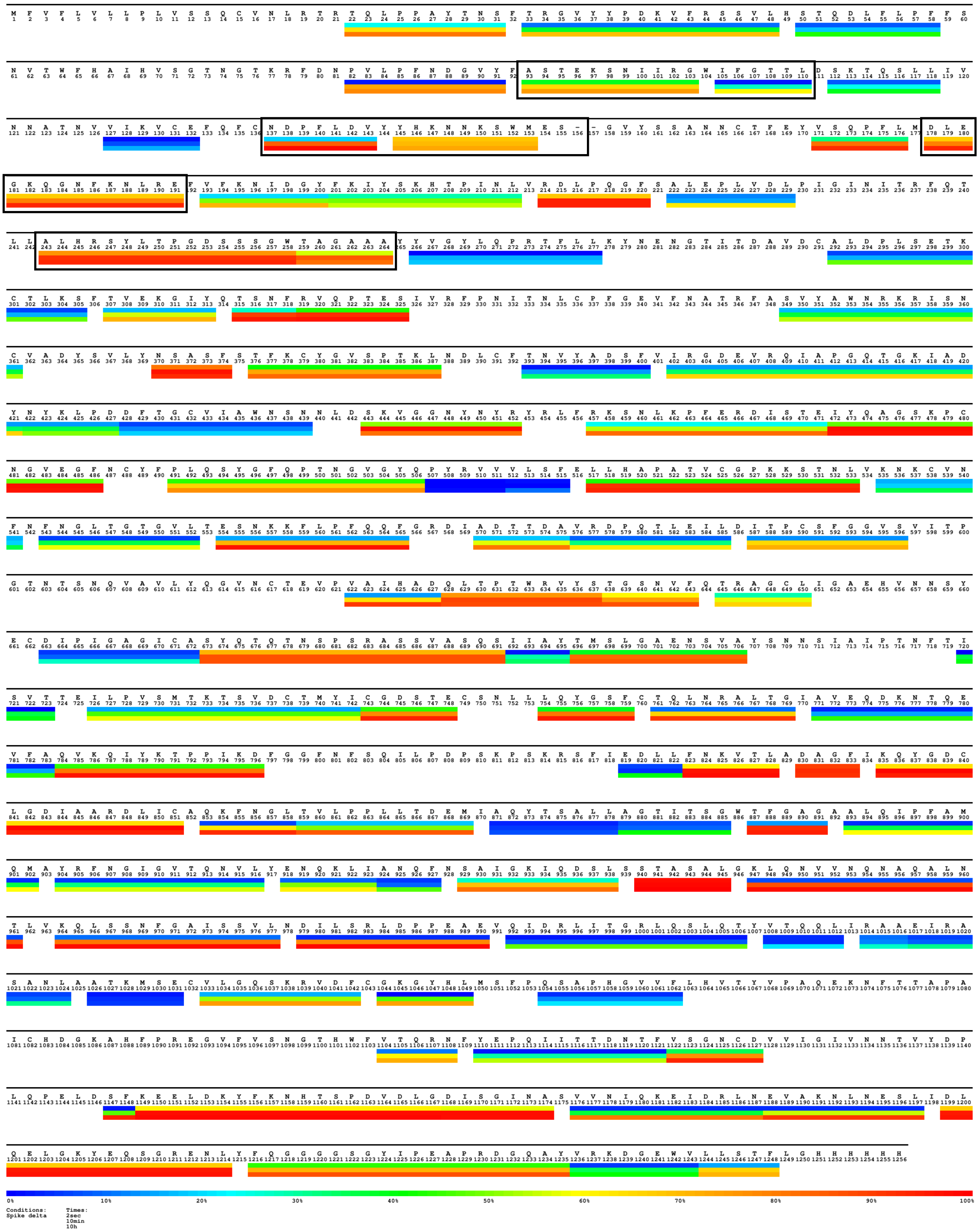

Supplementary Fig. 18. Heatmap of delta spike for time points 20 s on ice (~2 s at 23 °C), 10 min at 23 °C and 360 min at 28 °C (~10 h at 23 °C). Selected peptide segments are coloured according to the % of uptake relative to the maximally labelled control. Sequence was adapted to the residue numbering of G614 spike. Peptide segments manifesting increased HDX in the NTD of spike variants are framed in black. The figure was generated with MS Tools.

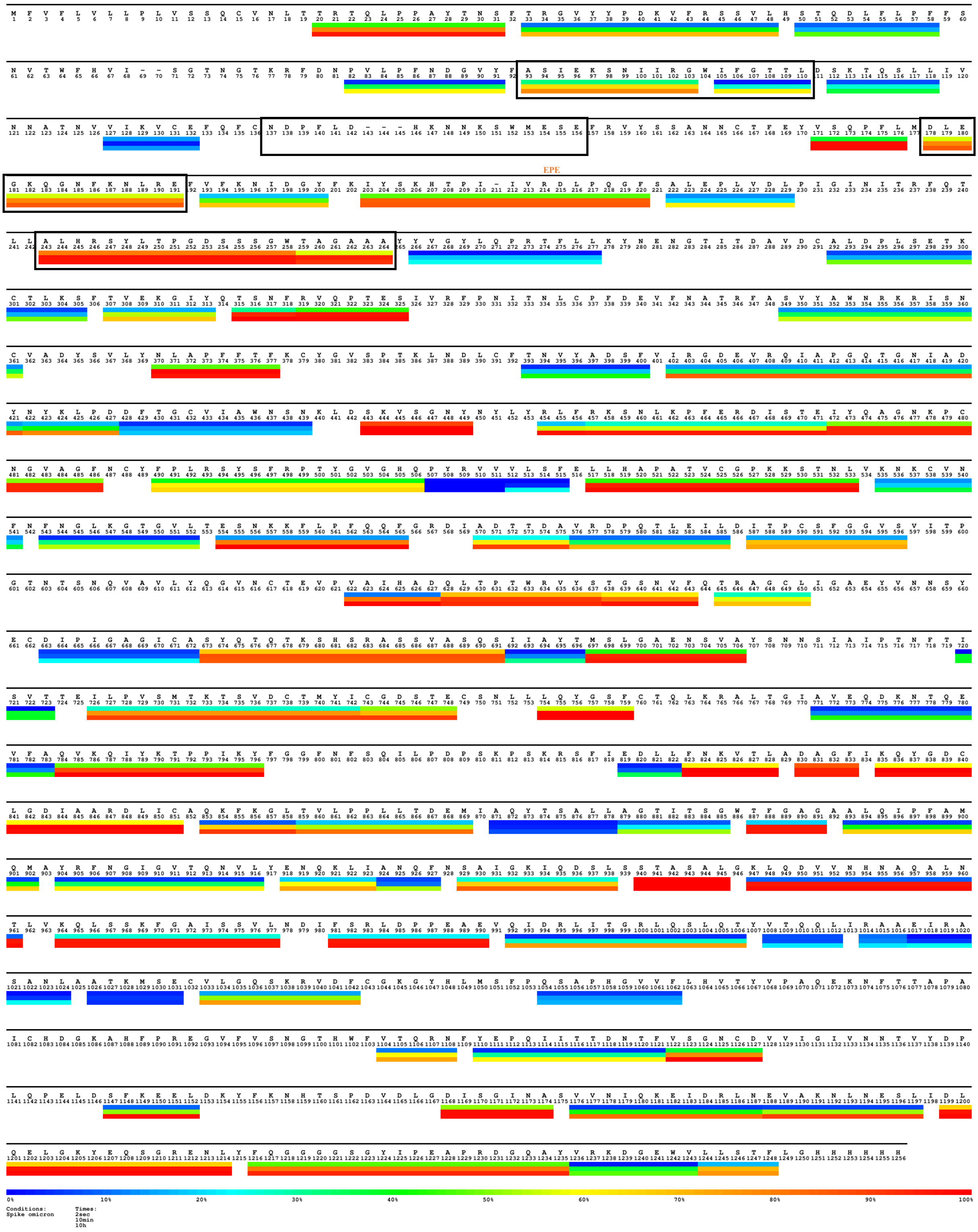

Supplementary Fig. 19. Heatmap of omicron spike for time points 20 s on ice (~2 s at 23 °C), 10 min at 23 °C and 360 min at 28 °C (~10 h at 23 °C). Selected peptide segments are coloured according to the % of uptake relative to the maximally labelled control. Sequence was adapted to the residue numbering of G614 spike. Peptide segments manifesting increased HDX in the NTD of spike variants are framed in black. Figure was generated with MS Tools.

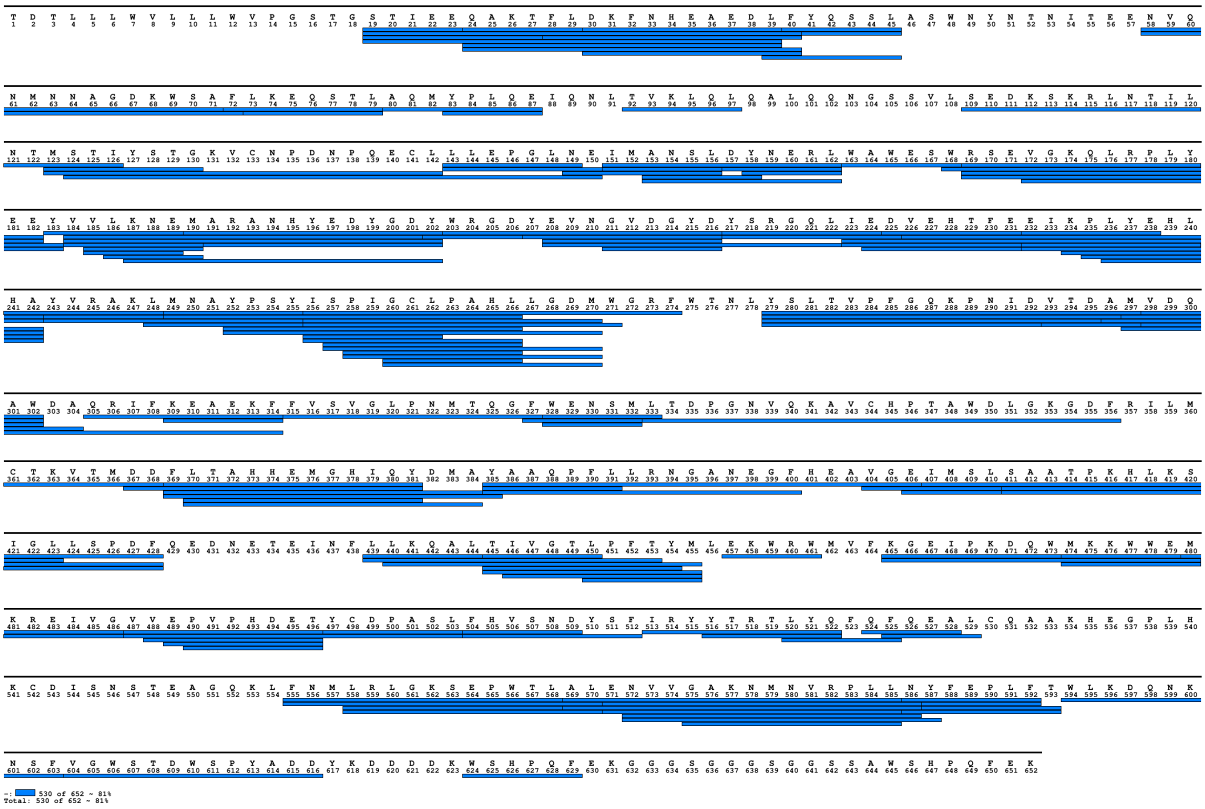

Supplementary Fig. 20. Coverage map of peptides whose HDX was followed for ACE2 in apo state and in complex with spikes. The signal peptide (residues 1-18) is indicated but not present in the protein analysed. The figure was generated with MS Tools.

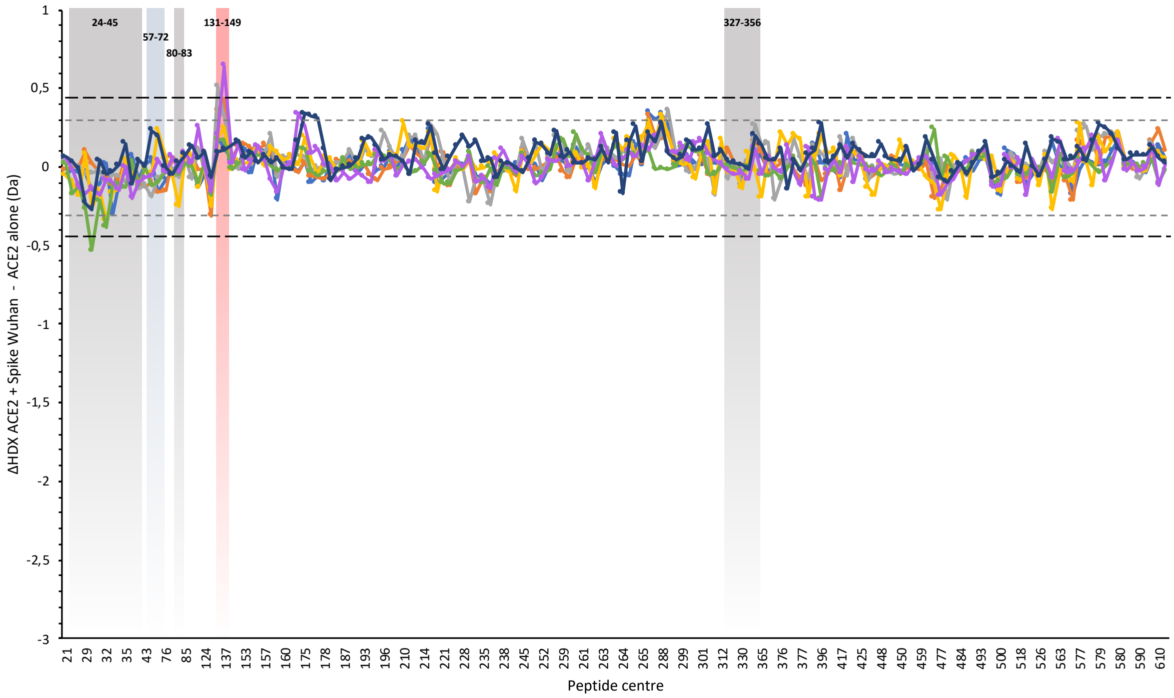

Supplementary Fig. 21. Difference plot illustrating the difference in HDX between ACE2 in complex with Wuhan spike and ACE2 alone (light blue line: 4 s on ice, orange line: 20 s on ice, grey line: 15 s at 23 °C, yellow line: 1 min at 23 °C, green line: 10 min at 23 °C, violet line: 100 min at 23 °C, dark blue line: 360 min at 28 °C). Peptide segments of interest are highlighted. The peptides are arranged according to their peptide centre residue. A dotted grey line indicates the 98% CI as a threshold for significance and a dotted black line the 99% CI as a threshold for significance. Source data are provided as a Source Data file.

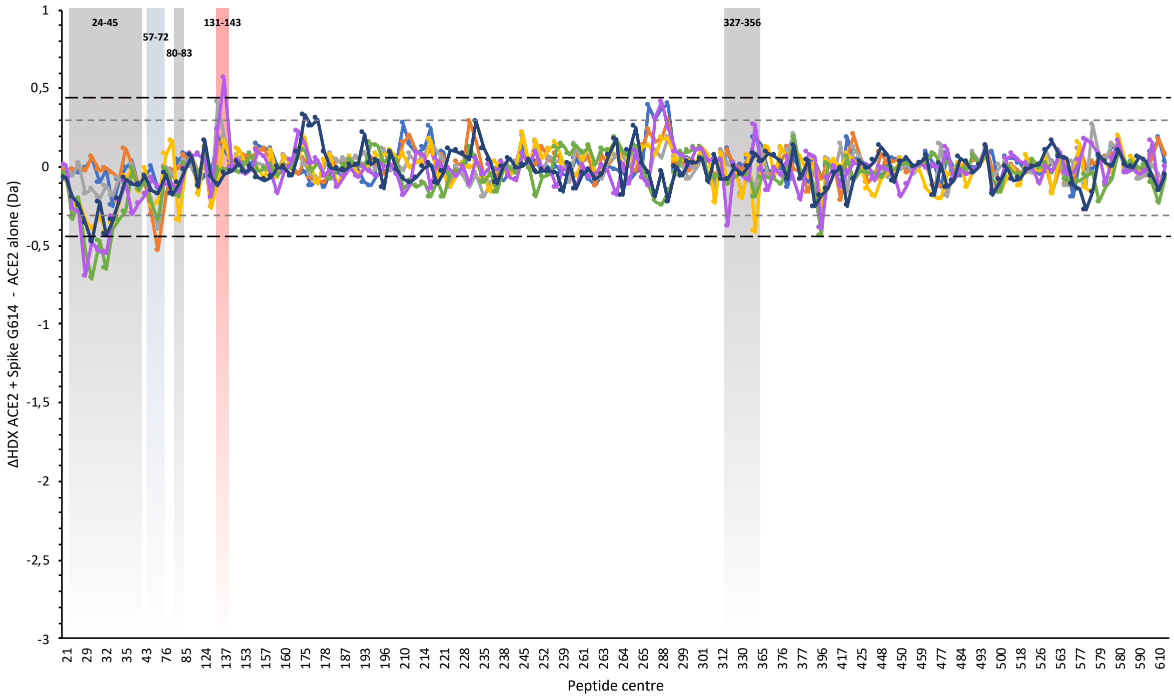

Supplementary Fig. 22. Difference plot illustrating the difference in HDX between ACE2 in complex with G614 spike and ACE2 alone (light blue line: 4 s on ice, orange line: 20 s on ice, grey line: 15 s at 23 °C, yellow line: 1 min at 23 °C, green line: 10 min at 23 °C, violet line: 100 min at 23 °C, dark blue line: 360 min at 28 °C). Peptide segments of interest are highlighted. The peptides are arranged according to their peptide centre residue. A dotted grey line indicates the 98% CI as a threshold for significance and a dotted black line the 99% CI as a threshold for significance. Source data are provided as a Source Data file.

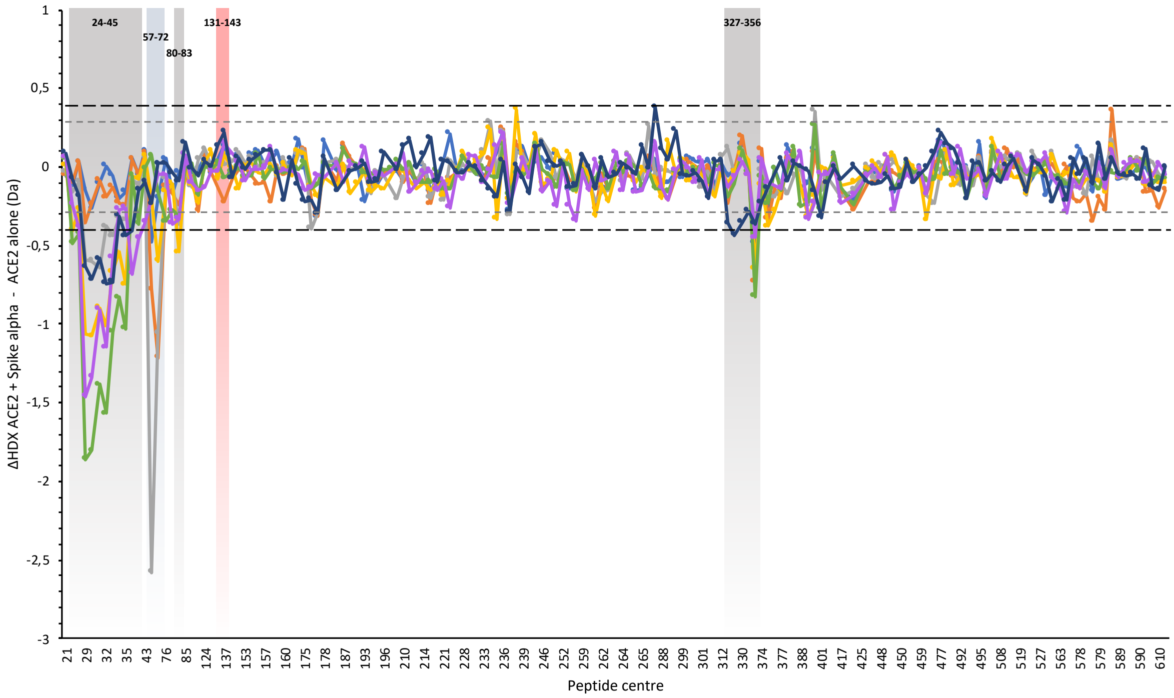

Supplementary Fig. 23. Difference plot illustrating the difference in HDX between ACE2 in complex with alpha spike and ACE2 alone (light blue line: 4 s on ice, orange line: 20 s on ice, grey line: 15 s at 23 °C, yellow line: 1 min at 23 °C, green line: 10 min at 23 °C, violet line: 100 min at 23 °C, dark blue line: 360 min at 28 °C). Peptide segments of interest are highlighted. The peptides are arranged according to their peptide centre residue. A dotted grey line indicates the 98% CI as a threshold for significance and a dotted black line the 99% CI as a threshold for significance. Source data are provided as a Source Data file.

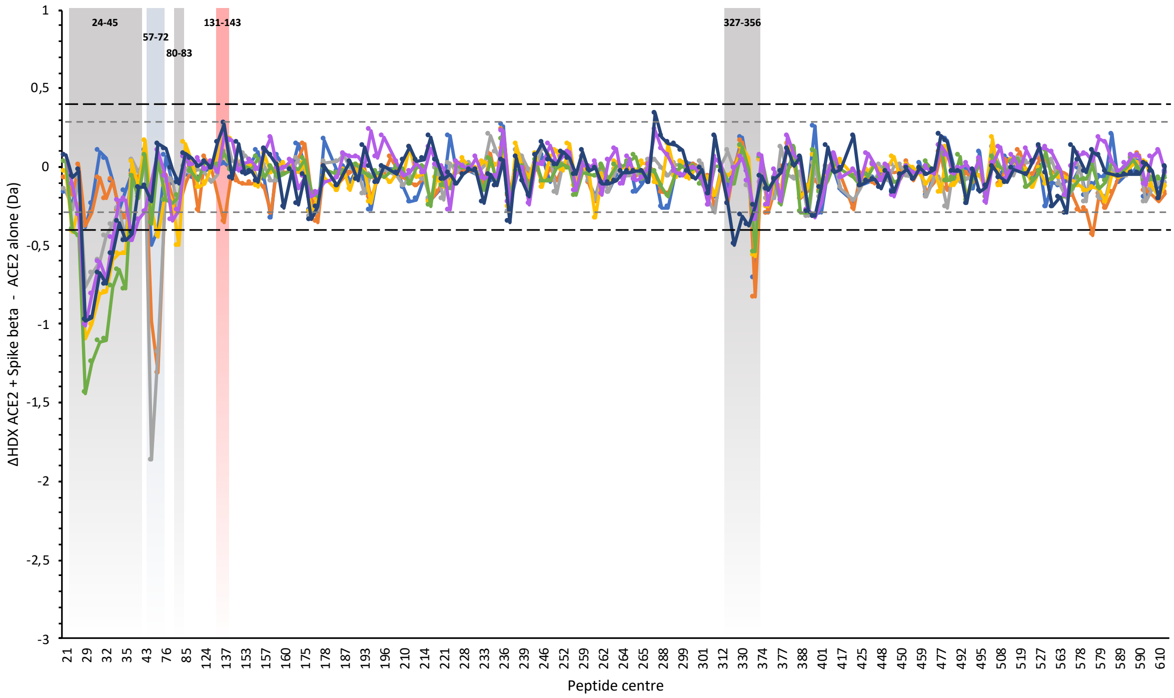

Supplementary Fig. 24. Difference plot illustrating the difference in HDX between ACE2 in complex with beta spike and ACE2 alone (light blue line: 4 s on ice, orange line: 20 s on ice, grey line: 15 s at 23 °C, yellow line: 1 min at 23 °C, green line: 10 min at 23 °C, violet line: 100 min at 23 °C, dark blue line: 360 min at 28 °C). Peptide segments of interest are highlighted. The peptides are arranged according to their peptide centre residue. A dotted grey line indicates the 98% CI as a threshold for significance and a dotted black line the 99% CI as a threshold for significance. Source data are provided as a Source Data file.

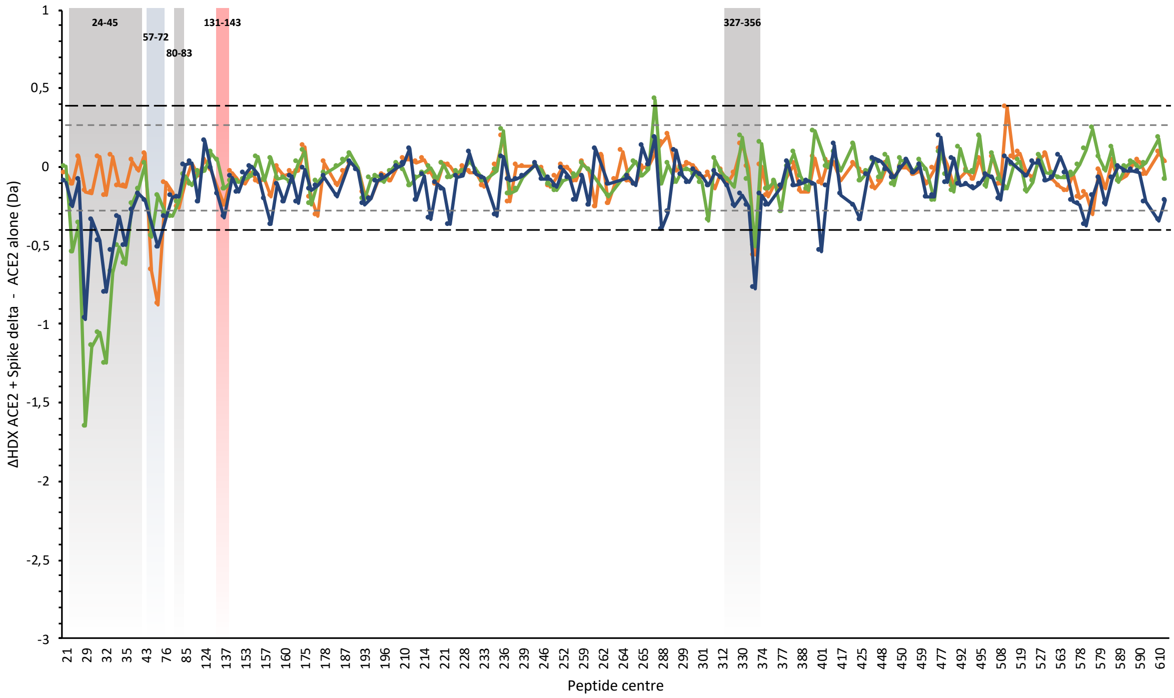

Supplementary Fig. 25. Difference plot illustrating the difference in HDX between ACE2 in complex with delta spike and ACE2 alone (orange line indicates: 20 s on ice, green line: 10 min at 23 °C, dark blue line: 360 min at 28 °C). Peptide segments of interest are highlighted. The peptides are arranged according to their peptide centre residue. A dotted grey line indicates the 98% CI as a threshold for significance and a dotted black line the 99% CI as a threshold for significance. Source data are provided as a Source Data file.

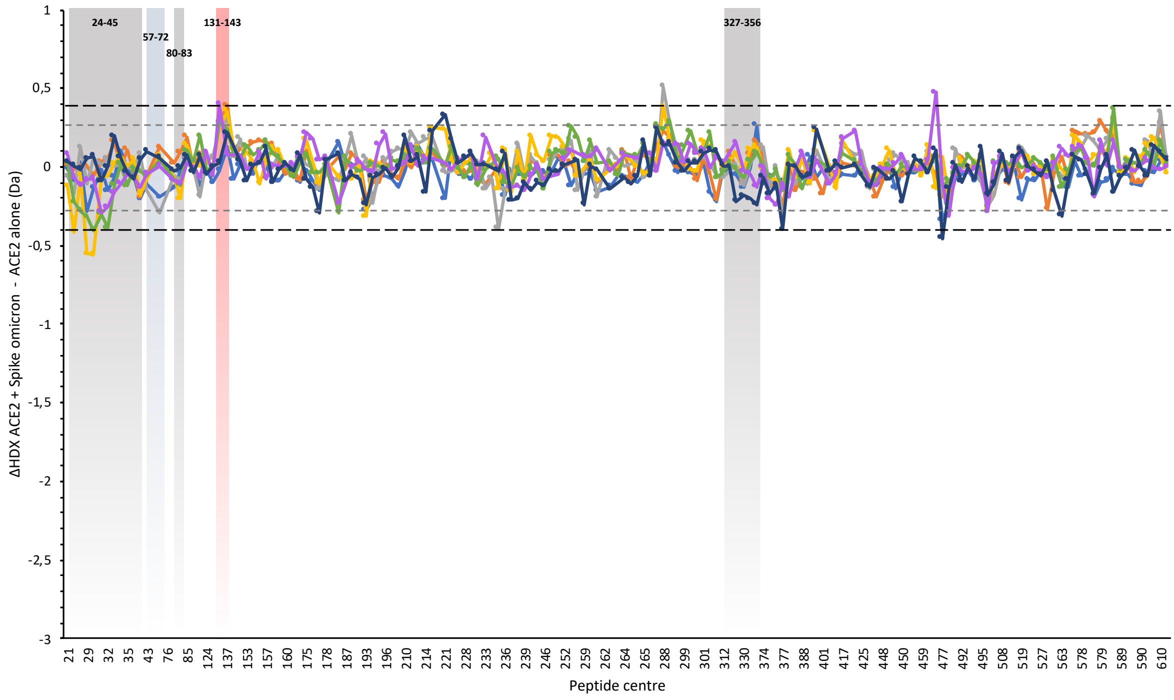

Supplementary Fig. 26. Difference plot illustrating the difference in HDX between ACE2 in complex with omicron spike and ACE2 alone (light blue line: 4 s on ice, orange line: 20 s on ice, grey line: 15 s at 23 °C, yellow line: 1 min at 23 °C, green line: 10 min at 23 °C, violet line: 100 min at 23 °C, dark blue line: 360 min at 28 °C). Peptide segments of interest are highlighted. The peptides are arranged according to their peptide centre residue. A dotted grey line indicates the 98% CI as a threshold for significance and a dotted black line the 99% CI as a threshold for significance. Source data are provided as a Source Data file.

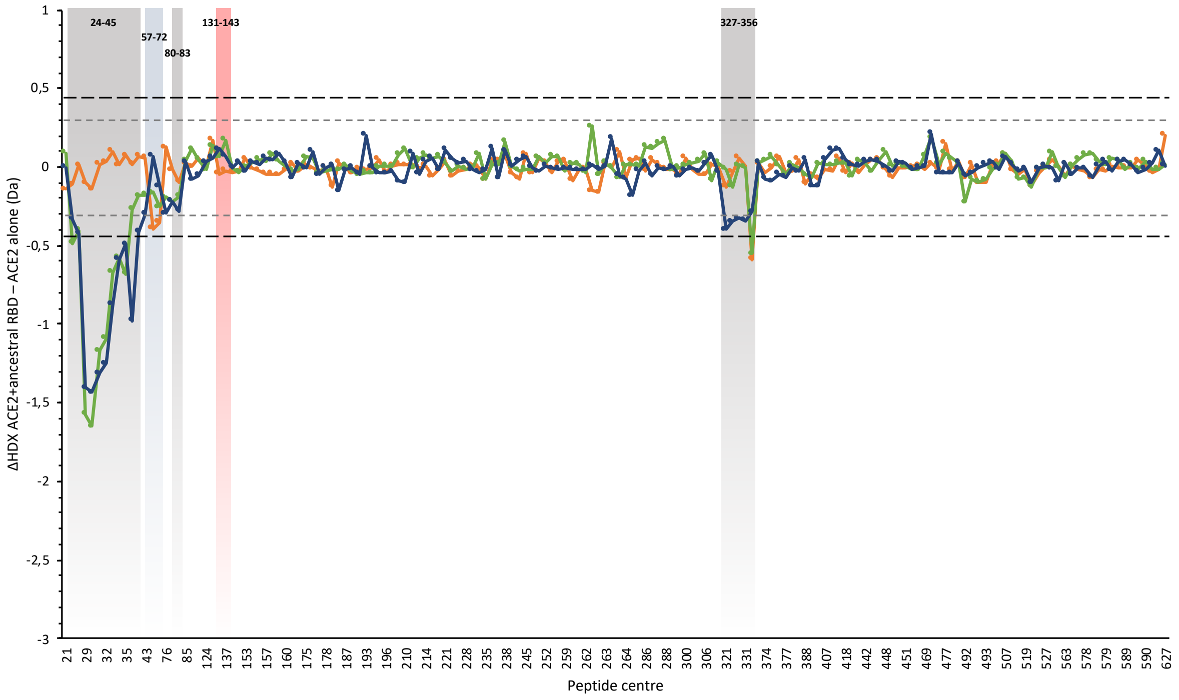

Supplementary Fig. 27. Difference plot illustrating the difference in HDX between ACE2 in complex with the isolated ancestral RBD and ACE2 alone (orange line indicates: 20 s on ice, green line: 10 min at 23 °C, dark blue line: 360 min at 28 °C). Peptide segments of interest are highlighted. The peptides are arranged according to their peptide centre residue. A dotted grey line indicates the 98% CI as a threshold for significance and a dotted black line the 99% CI as a threshold for significance. Source data are provided as a Source Data file.

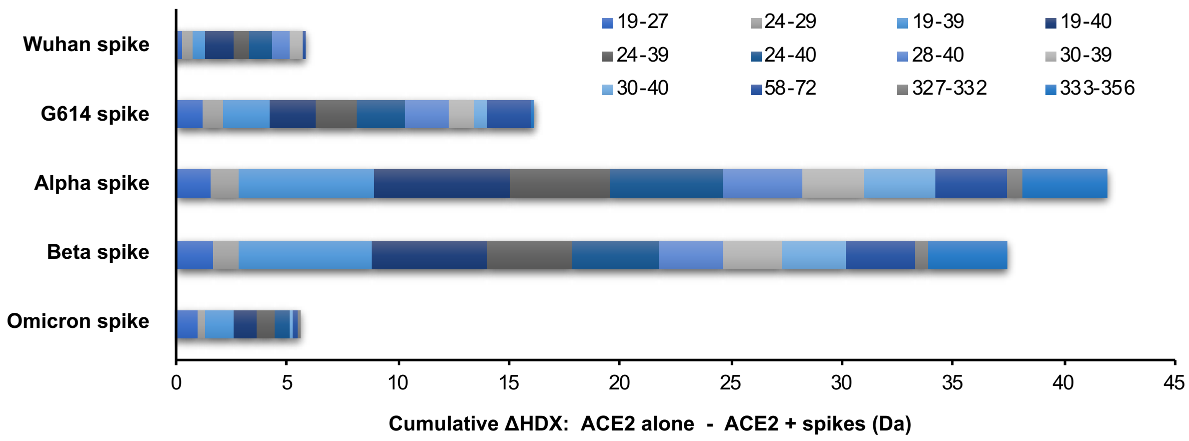

Supplementary Fig. 28. ACE2 receptor affinity and avidity of spikes. Extended plot of Fig. 3 of the main text. Plot illustrating cumulative differences in HDX (ΔHDX) between ACE2 and ACE2 in complex with spikes for selected peptides of interests through all time points (delta spike has been excluded as not all time points were sampled).

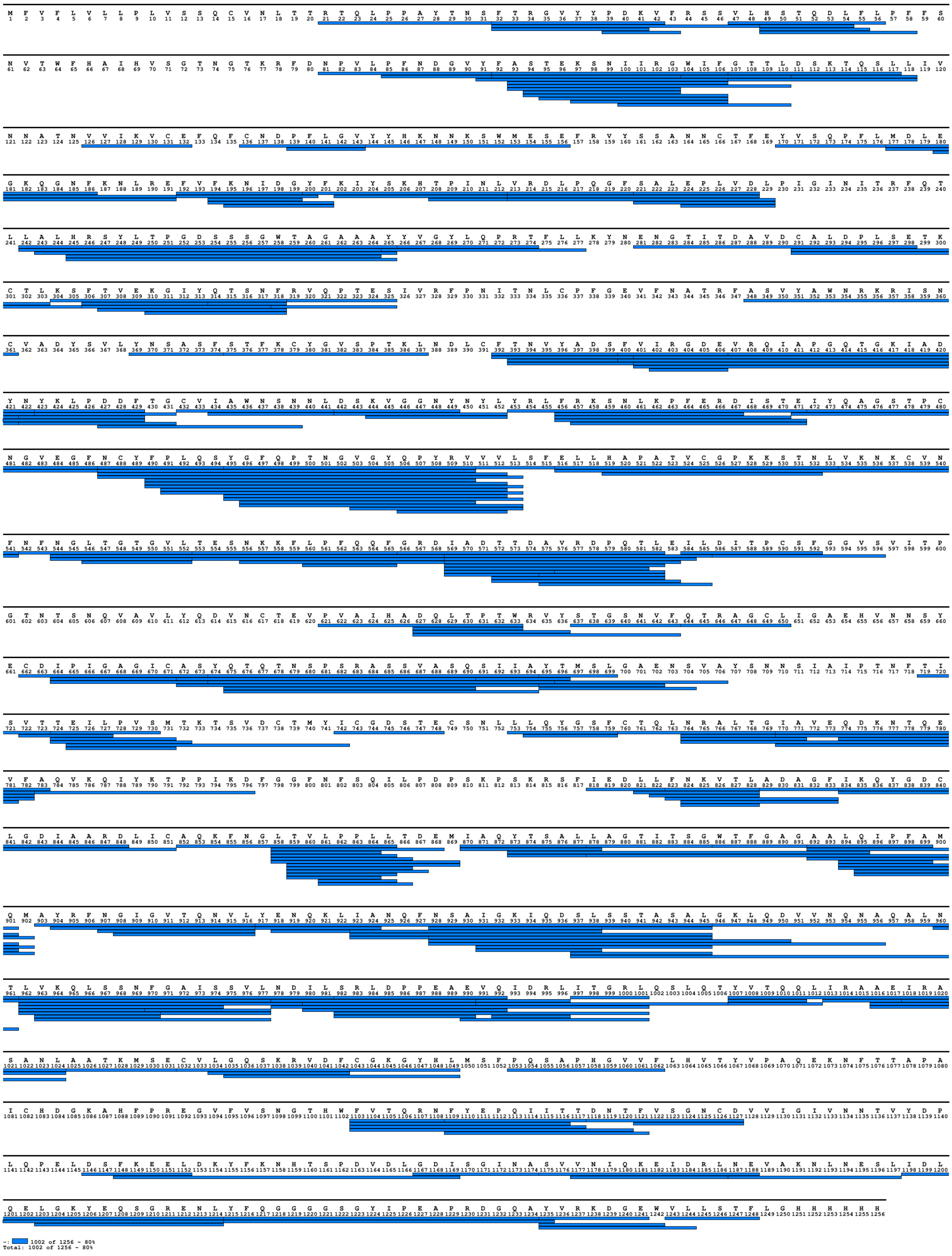

Supplementary Fig. 29. Coverage map of peptides whose HDX was followed for studying Wuhan/G614 spike-ACE2 complexes. The signal peptide (residues 1-13) is indicated but not present in the protein analysed. The figure was generated with MS Tools.

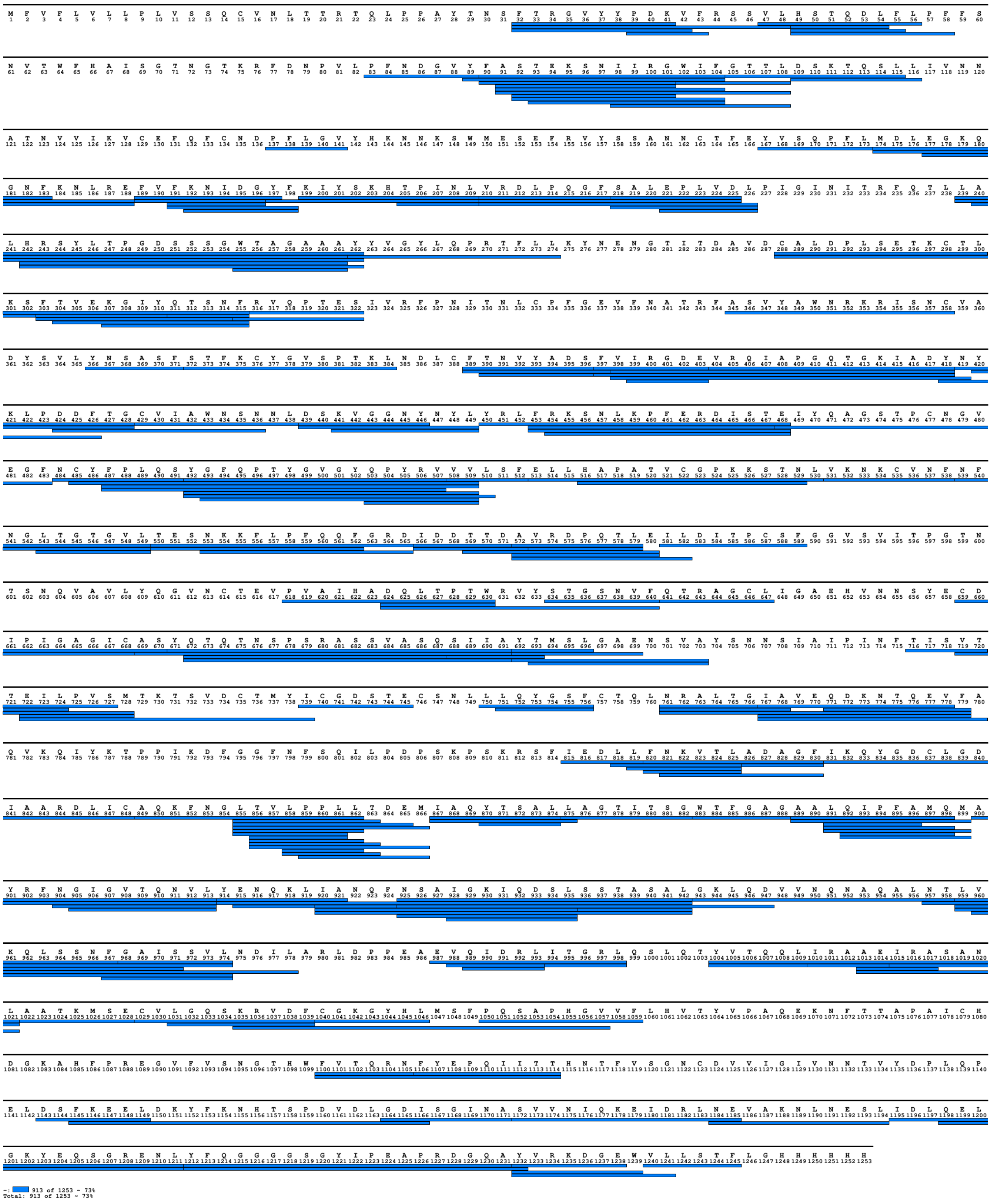

Supplementary Fig. 30. Coverage map of peptides whose HDX was followed for studying alpha spike-ACE2 complex. The signal peptide (residues 1-13) is indicated but not present in the protein analysed. The figure was generated with MS Tools.

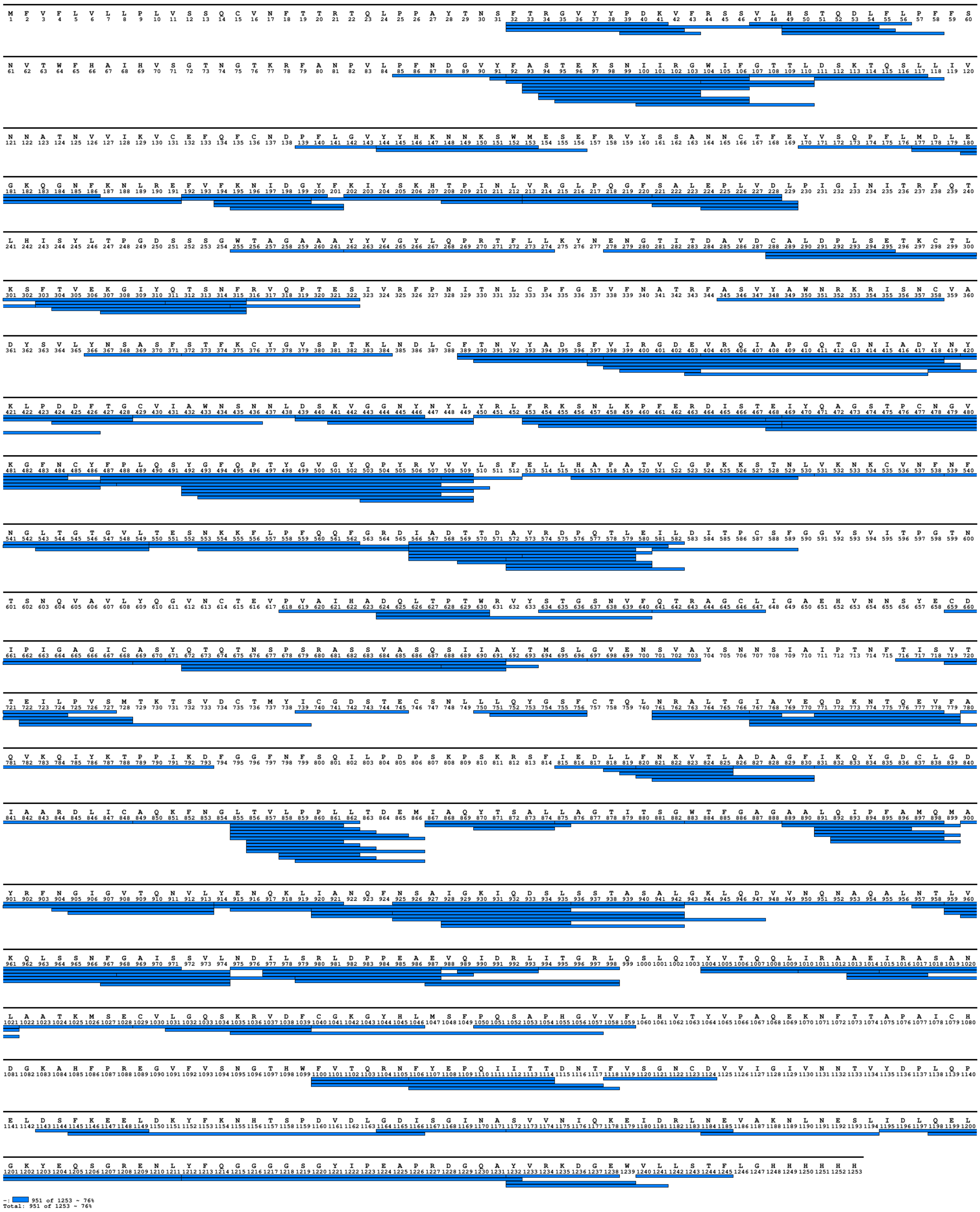

Supplementary Fig. 31. Coverage map of peptides whose HDX was followed for studying beta spike-ACE2 complex. The signal peptide (residues 1-13) is indicated but not present in the protein analysed. The figure was generated with MS Tools.

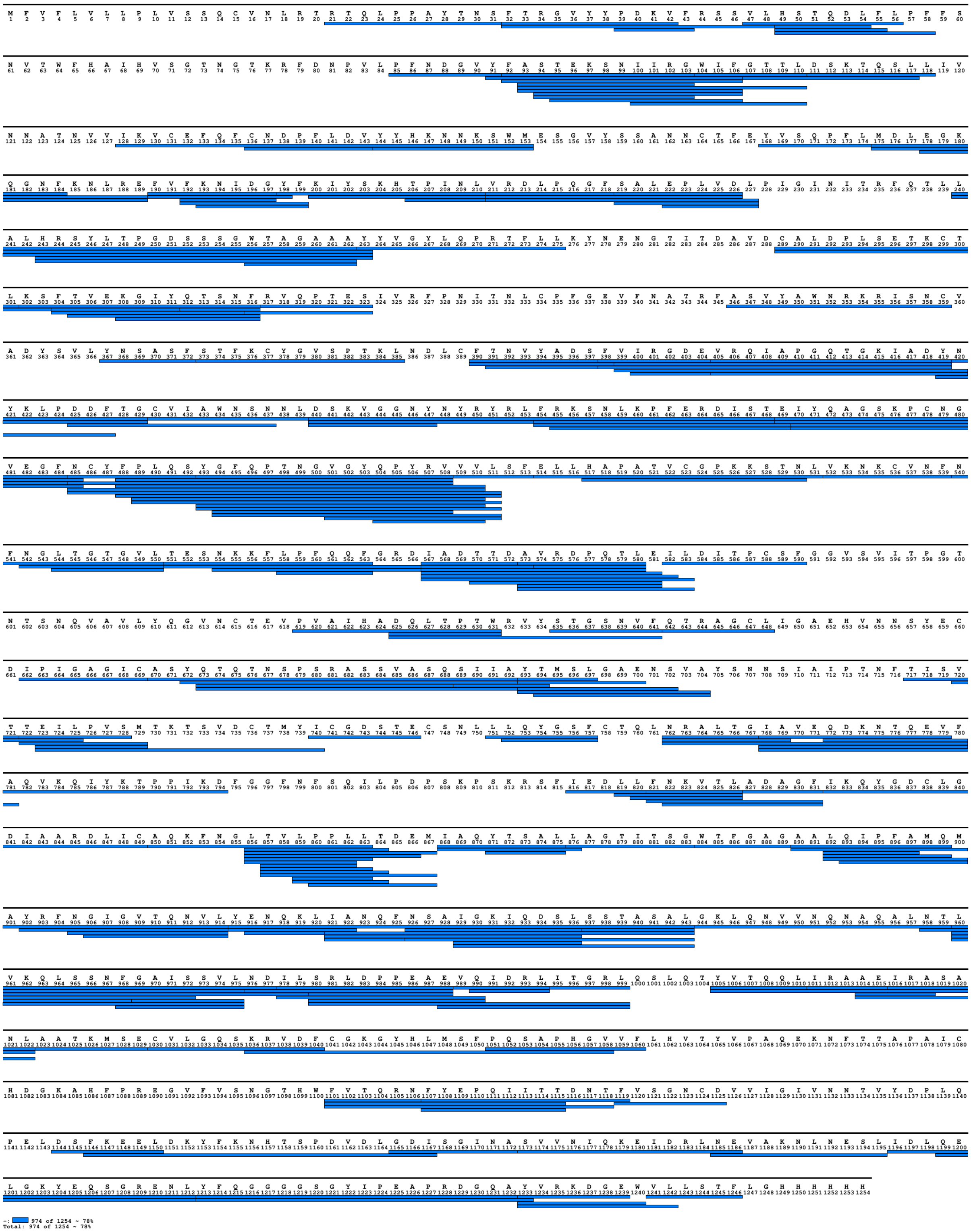

Supplementary Fig. 32. Coverage map of peptides whose HDX was followed for studying delta spike-ACE2 complex. The signal peptide (residues 1-13) is indicated but not present in the protein analysed. The figure was generated with MS Tools.

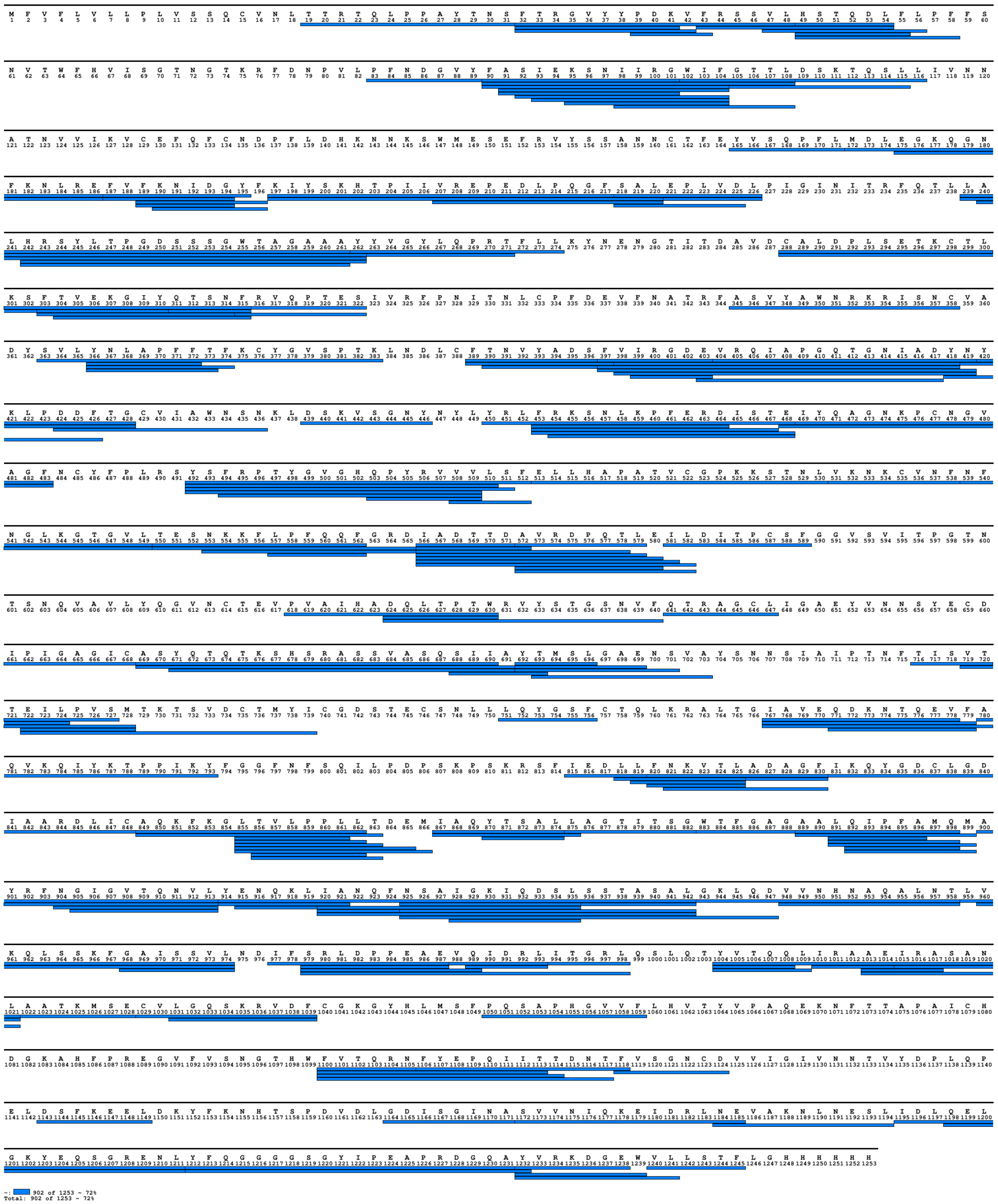

Supplementary Fig. 33. Coverage map of peptides whose HDX was followed for studying omicron spike-ACE2 complex. The signal peptide (residues 1-13) is indicated but not present in the protein analysed. The figure was generated with MS Tools.

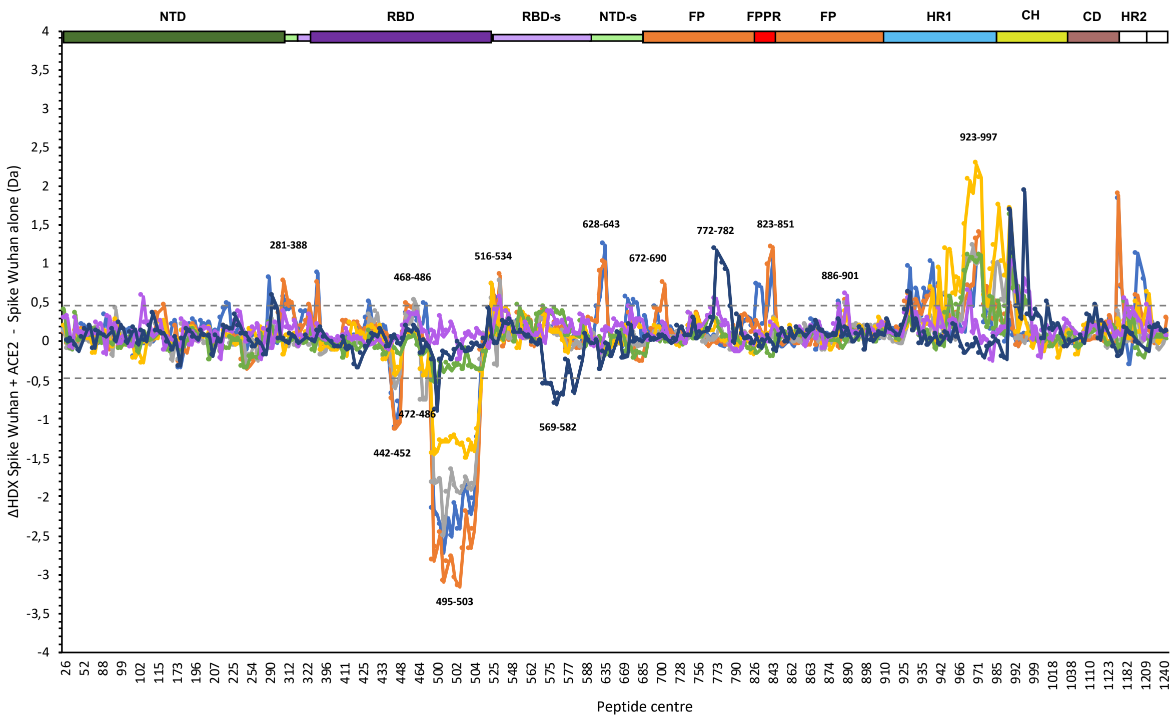

Supplementary Fig. 34. Difference plot illustrating the difference in HDX between Wuhan spike in complex with ACE2 and Wuhan spike alone (light blue line: 4 s on ice, orange line: 20 s on ice, grey line: 15 s at 23 °C, yellow line: 1 min at 23 °C, green line: 10 min at 23 °C, violet line: 100 min at 23 °C, dark blue line: 360 min at 28 °C). Residues comprising a region with significant differences in HDX are indicated. The peptides are arranged according to their peptide centre residue. A dotted grey line indicates the 98% CI as a threshold for significance. The top bar shows spike subdomains. Source data are provided as a Source Data file.

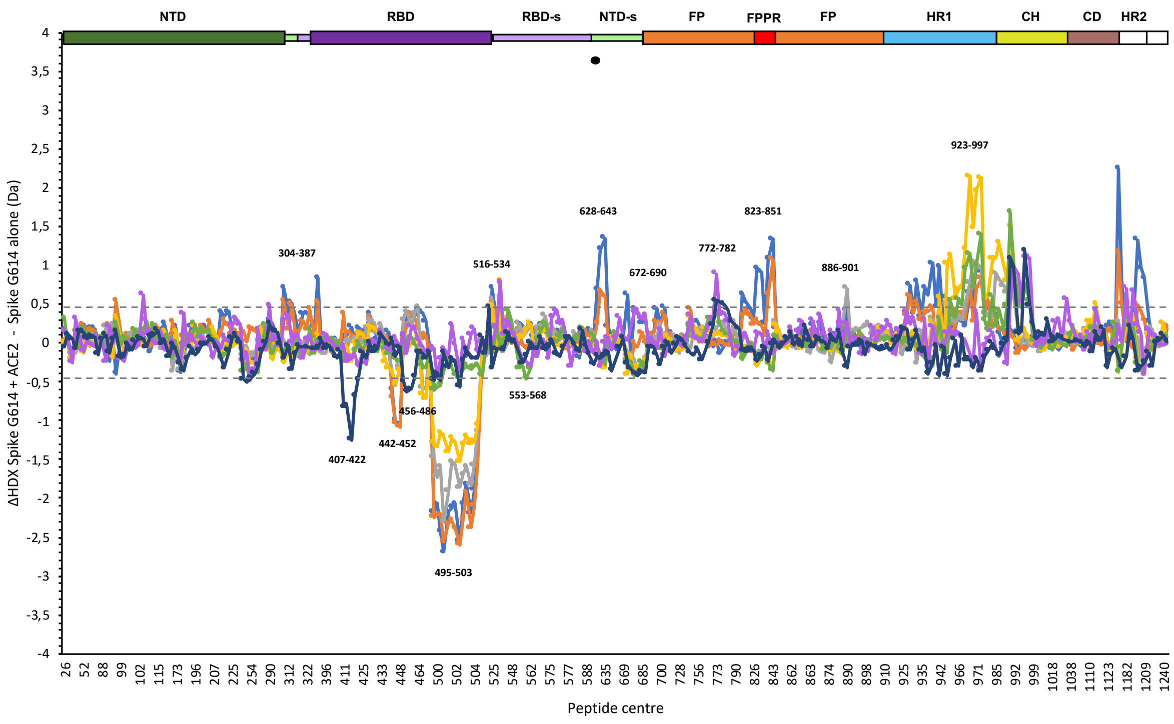

Supplementary Fig. 35. Difference plot illustrating the difference in HDX between G614 spike in complex with ACE2 and G614 spike alone (light blue line: 4 s on ice, orange line: 20 s on ice, grey line: 15 s at 23 °C, yellow line: 1 min at 23 °C, green line: 10 min at 23 °C, violet line: 100 min at 23 °C, dark blue line: 360 min at 28 °C). Residues comprising a region with significant differences in HDX are indicated. The peptides are arranged according to their peptide centre residue. A dotted grey line indicates the 98% CI as a threshold for significance. The top bar shows spike subdomains. The black dot indicates the position of D614G substitution. Source data are provided as a Source Data file.

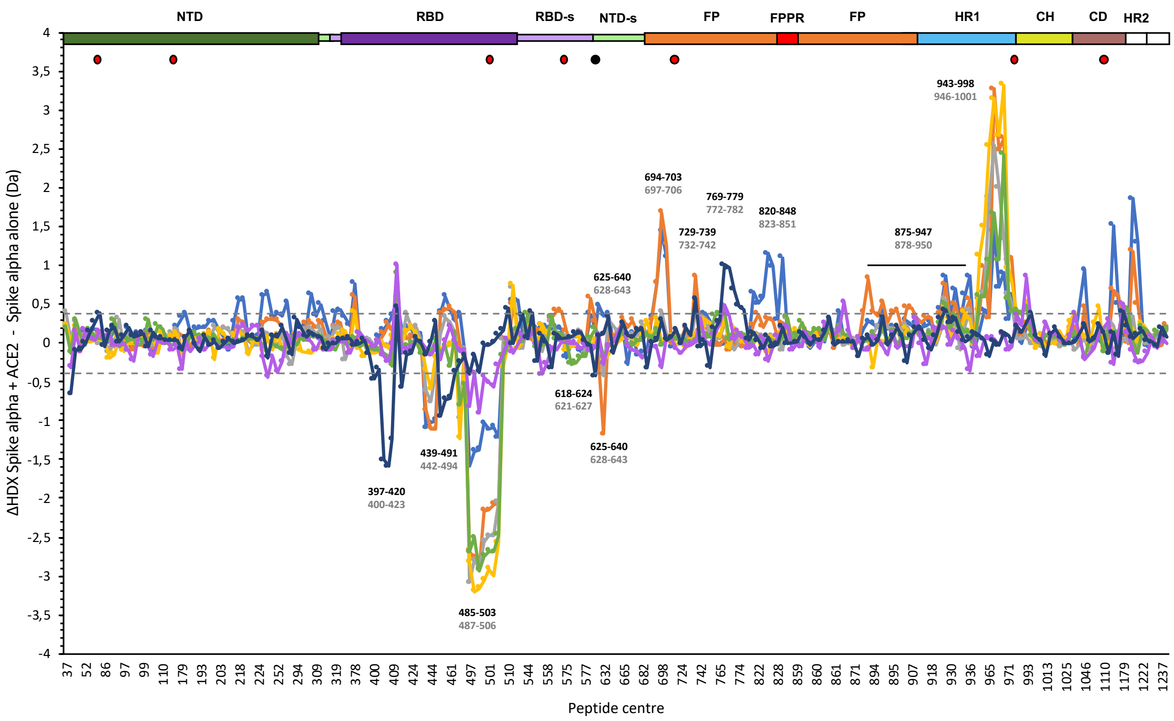

Supplementary Fig. 36. Difference plot illustrating the difference in HDX between alpha spike in complex with ACE2 and alpha spike alone (light blue line: 4 s on ice, orange line: 20 s on ice, grey line: 15 s at 23 °C, yellow line: 1 min at 23 °C, green line: 10 min at 23 °C, violet line: 100 min at 23 °C, dark blue line: 360 min at 28 °C). Residues comprising a region with significant differences in HDX are indicated (in black with residue numbers of alpha spike and in grey with residue numbers of G614 spike). The peptides are arranged according to their peptide centre residue. A dotted grey line indicates the 98% CI as a threshold for significance. The top bar shows spike subdomains. The black dot indicates the position of D614G substitution, whereas the red dots indicate the position of the other residue changes. Source data are provided as a Source Data file.

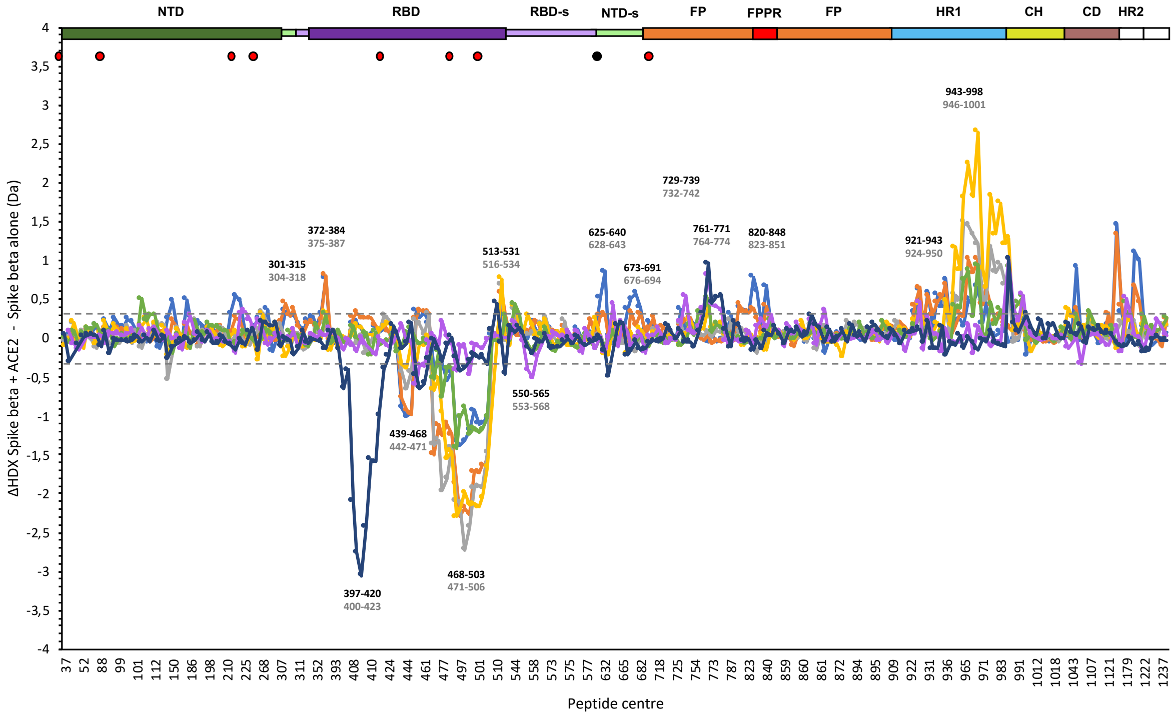

Supplementary Fig. 37. Difference plot illustrating the difference in HDX between beta spike in complex with ACE2 and beta spike alone (light blue line: 4 s on ice, orange line: 20 s on ice, grey line: 15 s at 23 °C, yellow line: 1 min at 23 °C, green line: 10 min at 23 °C, violet line: 100 min at 23 °C, dark blue line: 360 min at 28 °C). Residues comprising a region with significant differences in HDX are indicated (in black with residue numbers of beta spike and in grey with residue numbers of G614 spike). The peptides are arranged according to their peptide centre residue. A dotted grey line indicates the 98% CI as a threshold for significance. The top bar shows spike subdomains. The black dot indicates the position of D614G substitution, whereas the red dots indicate the position of the other residue changes. Source data are provided as a Source Data file.

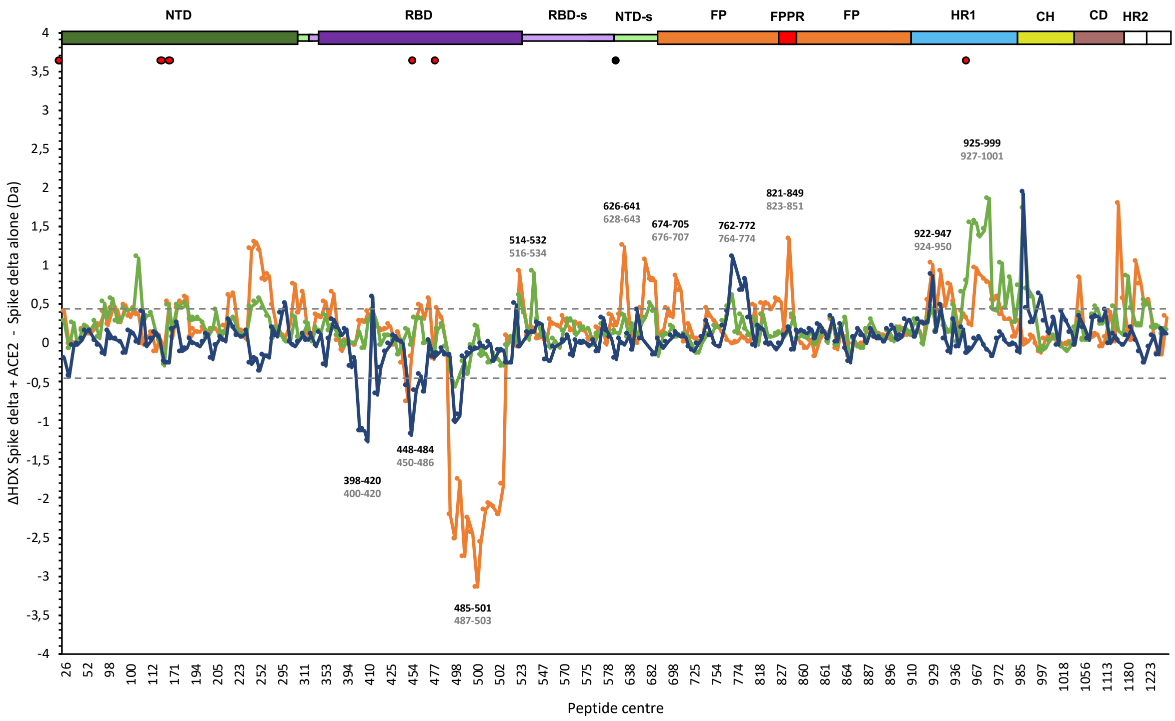

Supplementary Fig. 38. Difference plot illustrating the difference in HDX between delta spike in complex with ACE2 and delta spike alone (orange line: 20 s on ice, green line: 10 min at 23 °C, dark blue line: 360 min at 28 °C). Residues comprising a region with significant differences in HDX are indicated (in black with residue numbers of delta spike and in grey with residue numbers of G614 spike). The peptides are arranged according to their peptide centre residue. A dotted grey line indicates the 98% CI as a threshold for significance. The top bar shows spike subdomains. The black dot indicates the position of D614G substitution, whereas the red dots indicate the position of the other residue changes. Source data are provided as a Source Data file.

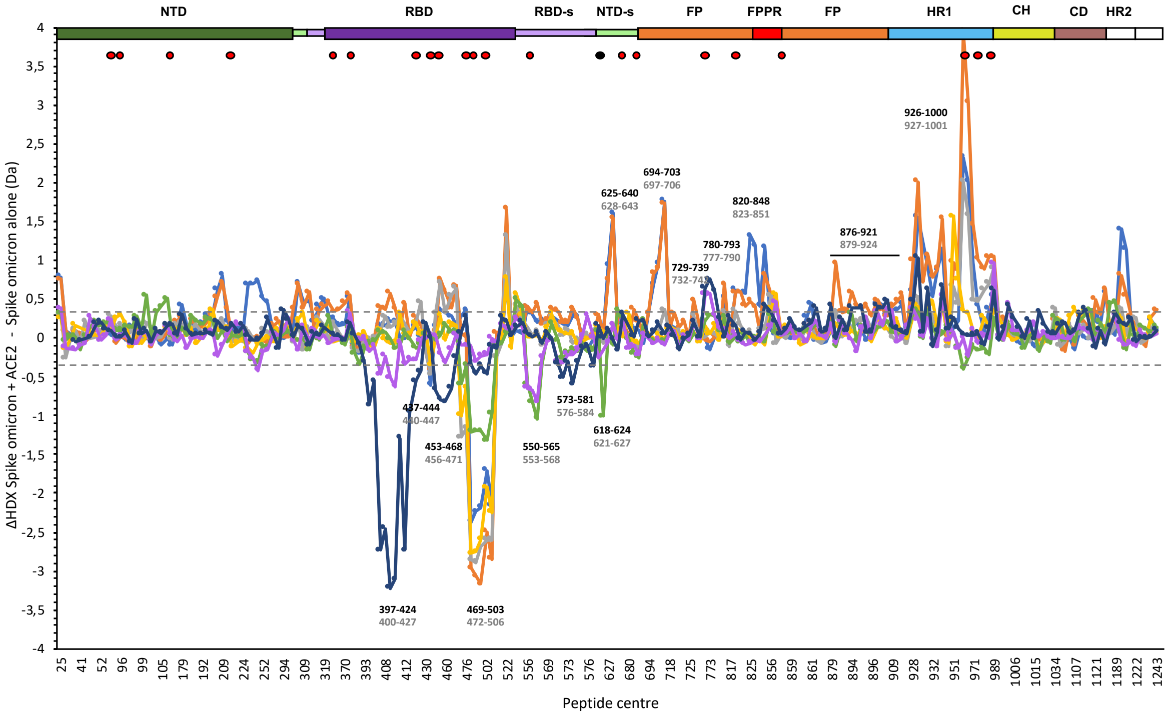

Supplementary Fig. 39. Difference plot illustrating the difference in HDX between omicron spike in complex with ACE2 and omicron spike alone (light blue line: 4 s on ice, orange line: 20 s on ice, grey line: 15 s at 23 °C, yellow line: 1 min at 23 °C, green line: 10 min at 23 °C, violet line: 100 min at 23 °C, dark blue line: 360 min at 28 °C). Residues comprising a region with significant differences in HDX are indicated (in black with residue numbers of omicron spike and in grey with residue numbers of G614 spike). The peptides are arranged according to their peptide centre residue. A dotted grey line indicates the 98% CI as a threshold for significance. The top bar shows spike subdomains. The black dot indicates the position of D614G substitution, whereas the red dots indicate the position of the other residue changes. Source data are provided as a Source Data file.

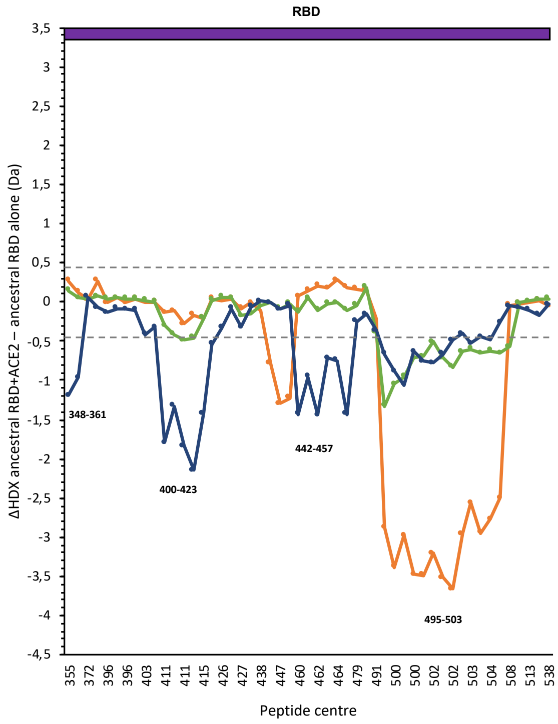

Supplementary Fig. 40. Difference plot illustrating the difference in HDX between the isolated ancestral RBD in complex with ACE2 and the isolated ancestral RBD alone (orange line: 20 s on ice, green line: 10 min at 23 °C, dark blue line: 360 min at 28 °C). Residues comprising a region with significant differences in HDX are indicated. The peptides are arranged according to their peptide centre residue. A dotted grey line indicates the 98% CI as a threshold for significance. Source data are provided as a Source Data file.

a

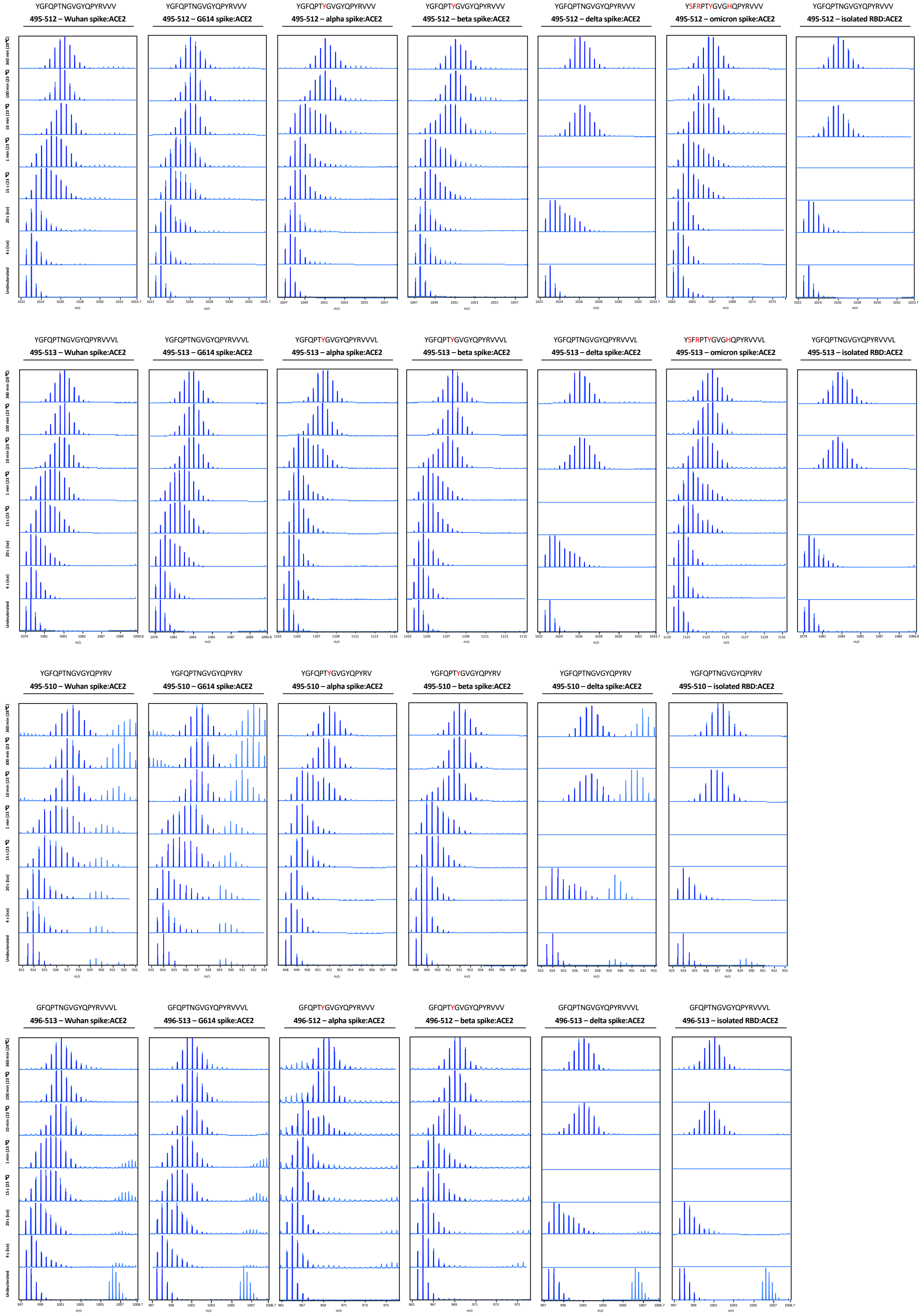
YGFQPTNGVGYQPYRVVV
495-512 – Wuhan spike:ACE2
YGFQPTNGVGYQPYRVVV
495-512 – G614 spike:ACE2
YGFQPTYGVGYQPYRVVV
495-512 – alpha spike:ACE2
YGFQPTYGVGYQPYRVVV
495-512 – beta spike:ACE2
YGFQPTNGVGYQPYRVVV
495-512 – delta spike:ACE2
YSFRPTYGVGHQPYRVVV
495-512 – omicron spike:ACE2
YGFQPTNGVGYQPYRVVV
495-512 – isolated RBD:ACE2
YGFQPTNGVGYQPYRVVVL
495-513 – Wuhan spike:ACE2
YGFQPTNGVGYQPYRVVVL
495-513 – G614 spike:ACE2
YGFQPTYGVGYQPYRVVVL
495-513 – alpha spike:ACE2
YGFQPTYGVGYQPYRVVVL
495-513 – beta spike:ACE2
YGFQPTNGVGYQPYRVVVL
495-513 – delta spike:ACE2
YSFRPTYGVGHQPYRVVVL
495-513 – omicron spike:ACE2
YGFQPTNGVGYQPYRVVVL
495-513 – isolated RBD:ACE2
YGFQPTNGVGYQPYRV
495-510 – Wuhan spike:ACE2
YGFQPTNGVGYQPYRV
495-510 – G614 spike:ACE2
YGFQPTYGVGYQPYRV
495-510 – alpha spike:ACE2
YGFQPTYGVGYQPYRV
495-510 – beta spike:ACE2
YGFQPTNGVGYQPYRV
495-510 – delta spike:ACE2
YGFQPTNGVGYQPYRV
495-510 – isolated RBD:ACE2
GFQPTNGVGYQPYRVVVL
496-513 – Wuhan spike:ACE2
GFQPTNGVGYQPYRVVVL
496-513 – G614 spike:ACE2
GFQPTYGVGYQPYRVVV
496-512 – alpha spike:ACE2
GFQPTYGVGYQPYRVVV
496-512 – beta spike:ACE2
GFQPTNGVGYQPYRVVVL
496-513 – delta spike:ACE2
GFQPTNGVGYQPYRVVVL
496-513 – isolated RBD:ACE2

b

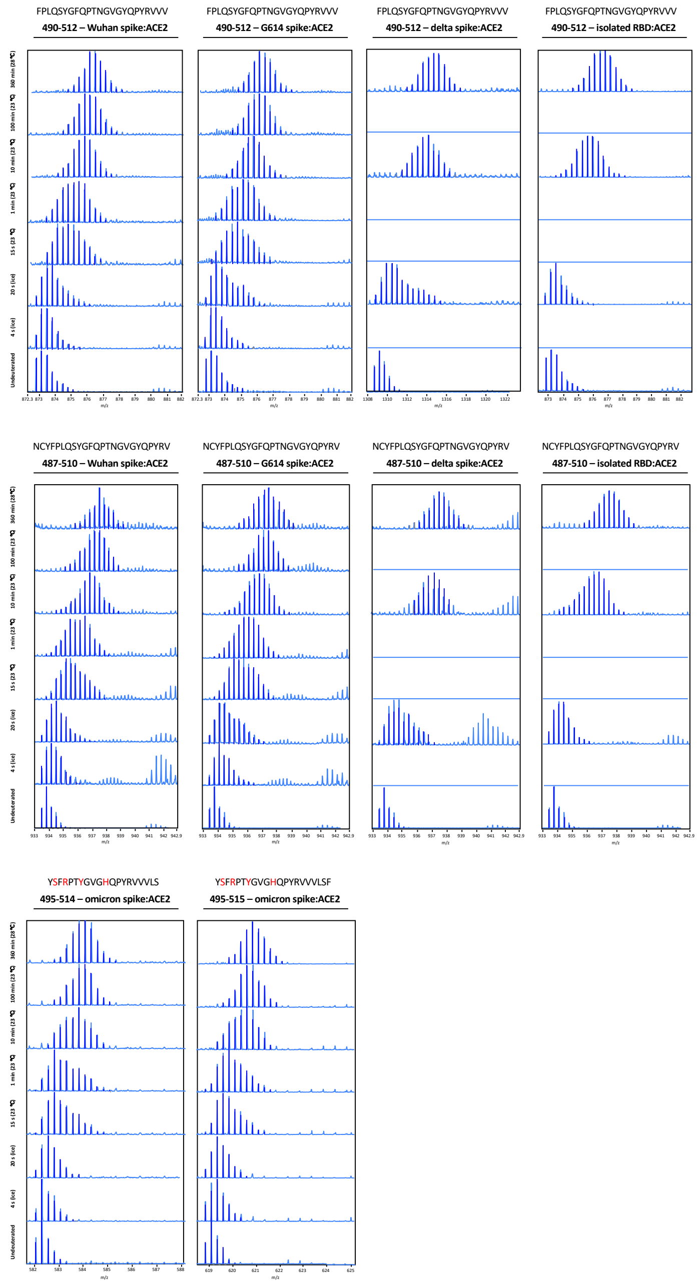

Supplementary Fig. 41 (a,b). Bimodal HDX profiles in selected peptides spanning residues 495-503 of spike receptor binding motif (RBM) of spike trimers and the isolated RBD in complex with ACE2. Stacked spectral plots elucidate the evolution of the bimodal isotopic envelopes across the time points studied and for different overlapping peptides in spike trimers. The bimodal isotopic envelopes of peptides 495-510 and 495-513 have been also analysed with HX-Express2[2] (see Supplementary Figs. 42-45). The isotopic envelopes of the isolated ancestral RBD does not display bimodal features nor evident peak broadening.

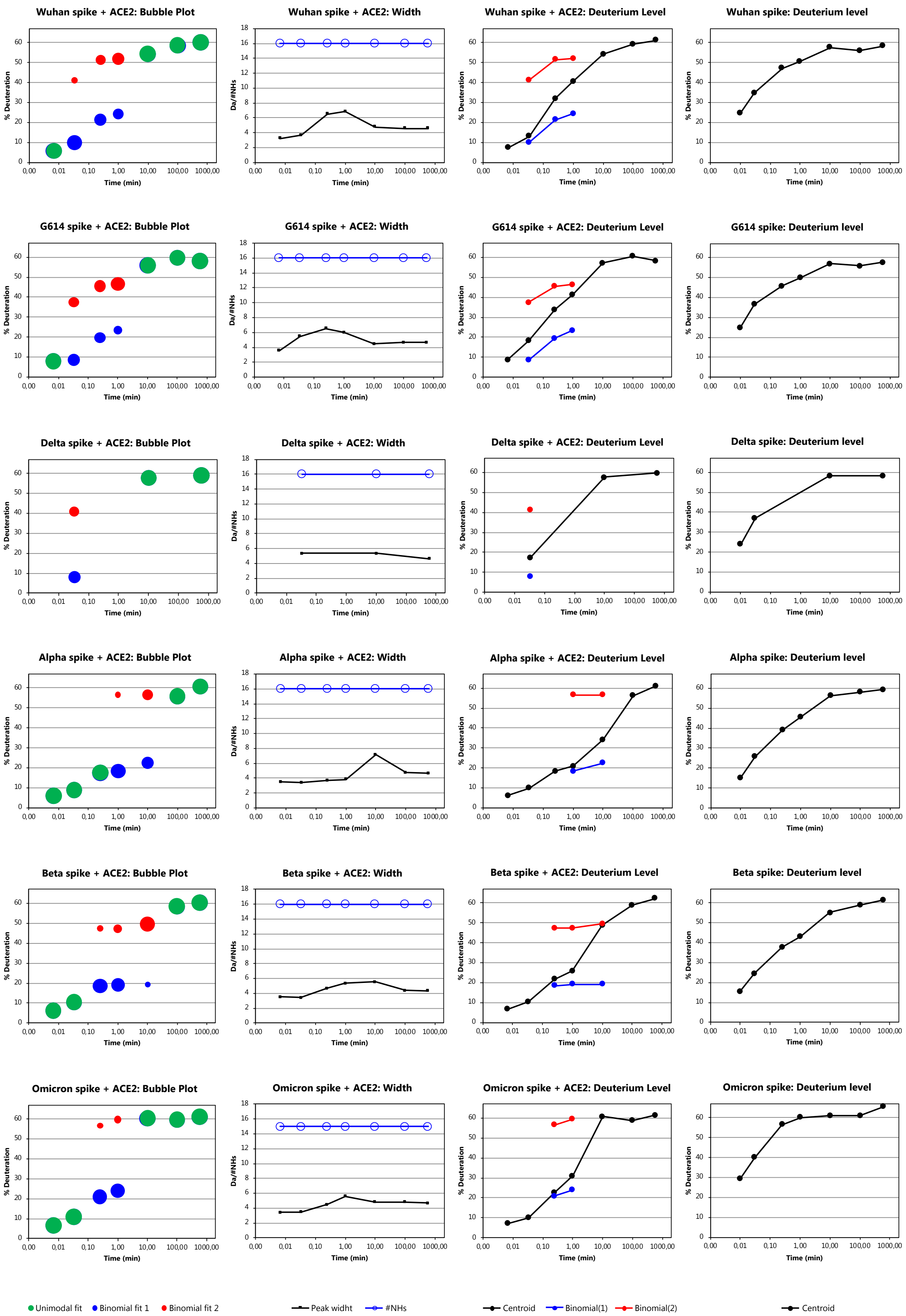
Wuhan spike + ACE2: Bubble Plot
Wuhan spike + ACE2: Width
Wuhan spike + ACE2: Deuterium Level
Wuhan spike: Deuterium level
G614 spike + ACE2: Bubble Plot
G614 spike + ACE2: Width
G614 spike + ACE2: Deuterium Level
G614 spike: Deuterium level
Delta spike + ACE2: Bubble Plot
Delta spike + ACE2: Width
Delta spike + ACE2: Deuterium Level
Delta spike: Deuterium level
Alpha spike + ACE2: Bubble Plot
Alpha spike + ACE2: Width
Alpha spike + ACE2: Deuterium Level
Alpha spike: Deuterium level
Beta spike + ACE2: Bubble Plot
Beta spike + ACE2: Width
Beta spike + ACE2: Deuterium Level
Beta spike: Deuterium level
Omicron spike + ACE2: Bubble Plot
Omicron spike + ACE2: Width
Omicron spike + ACE2: Deuterium Level
Omicron spike: Deuterium level
% Deuteration
Da/#NHs
Time (min)
0,00
0,01
0,10
1,00
10,00
100,00
1000,00
Unimodal fit
Binomial fit 1
Binomial fit 2
Peak widht
#NHs
Centroid
Binomial(1)
Binomial(2)
Centroid

Supplementary Fig. 42. Analysis of the bimodal HDX profiles in the receptor binding motif (RBM) of spikes in complex with ACE2. From top to bottom: peptide YGFQPTNGVGYQPYRVVVL (495-513) of Wuhan spike; peptide YGFQPTNGVGYQPYRVVVL (495-513) of G614 spike; peptide YGFQPTNGVGYQPYRVVVL (493-511) of delta spike; peptide YGFQPTYGVGYQPYRVVVL (492-510) of alpha spike; peptide YGFQPTYGVGYQPYRVVVL (492-510) of beta spike; peptide YSFRPTYGVGHQPYRVVV (492-509) of omicron spike. From left to right: bubble plots providing an approximation of the level of deuteration and the relative intensity of the low- and high-mass envelope (binomial fit 1 and binomial fit 2, respectively) for bimodal isotopic distributions and the level of deuteration of the unimodal envelope (unimodal fit), set at 100% intensity, in the ACE2 bound state; peak width of the isotopic distribution (calculated at 50% of Baseline Peak Intensity - BPI) and number of exchangble amides (#NHs), in the bound state; deuterium level of the low-mass envelopes (binomial 1), high-mass envelopes (binomial 2) and unimodal distributions (centroid) in ACE2-bound spike; deuterium level of spikes in apo state (unimodal distributions – centroid). Spectral analysis was conducted with HX-Express2[2] and graphs were authomatically generated upon fitting the isotopic envelopes shown in Supplementary Fig. 43. Bimodal fit was applied to peptide spectra at time points showing clear features of bimodality and/or enhanced peak width. The size of the two populations approximated with bimodal fit are reported in Fig. 5 of the main text. These data are indicative and semi-quantitative and need to be regarded as trends. Note that the high-mass population does not manifest at early time points, thus does not follow the HDX of the unbound state.

a

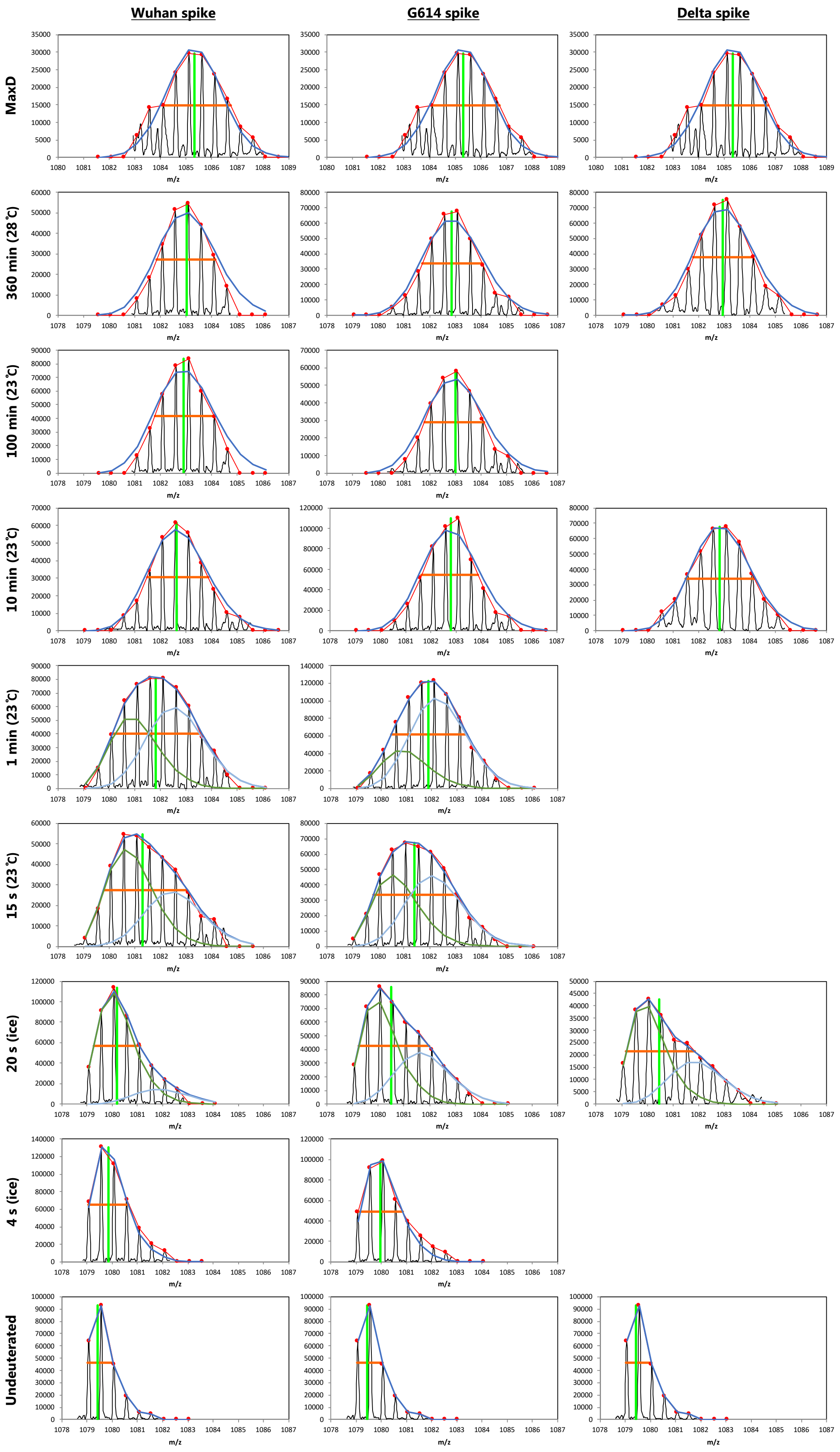

Wuhan spike
G614 spike
Delta spike
MaxD
360 min (28 ̊C)
100 min (23 ̊C)
10 min (23 ̊C)
1 min (23 ̊C)
15 s (23 ̊C)
20 s (ice)
4 s (ice)
Undeuterated
m/z

b

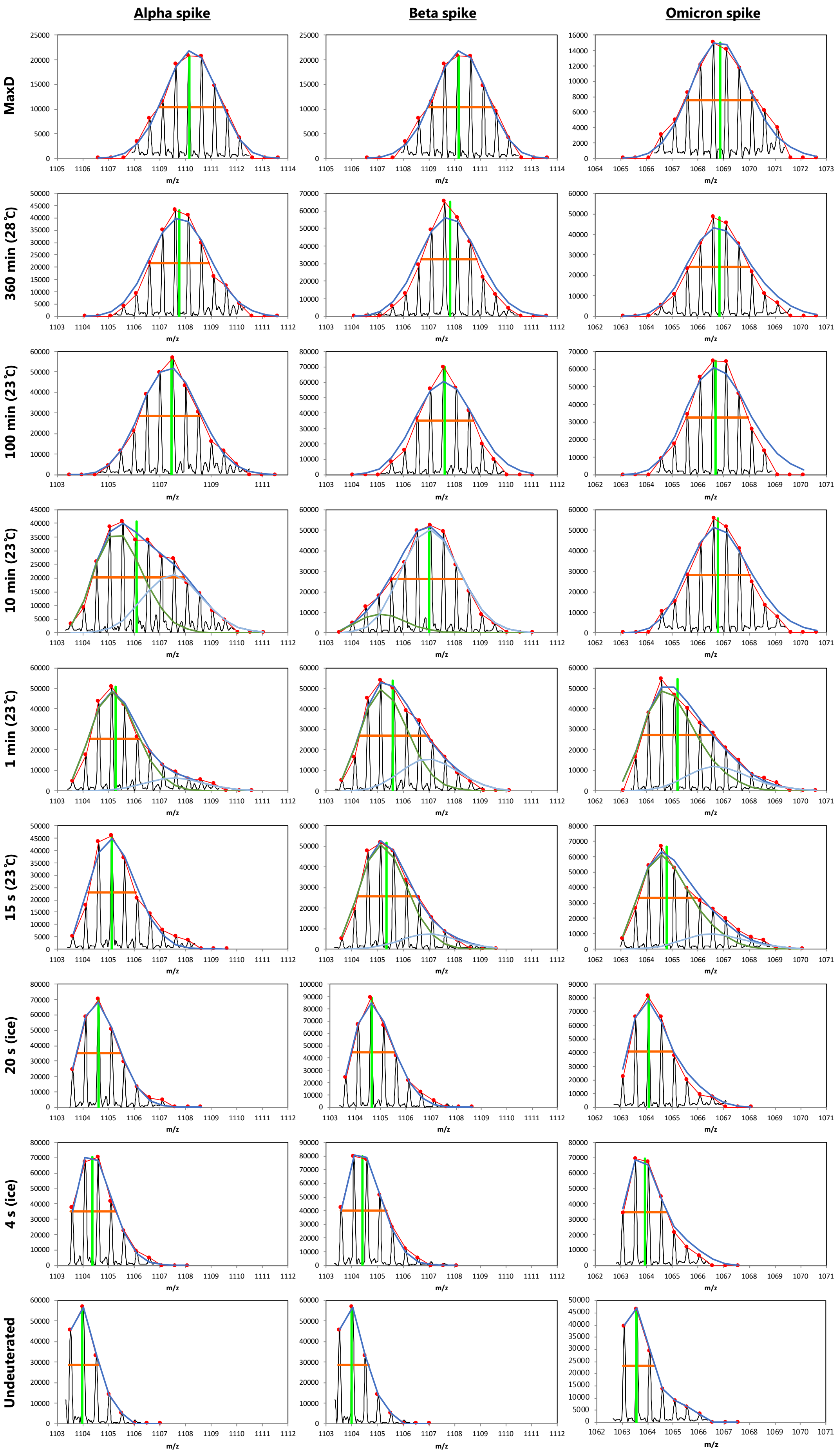
Alpha spike
Beta spike
Omicron spike
MaxD
360 min (28℃)
100 min (23℃)
10 min (23℃)
1 min (23℃)
15 s (23℃)
20 s (ice)
4 s (ice)
Undeuterated
m/z

Supplementary Fig. 43. Bimodal HDX profiles in the receptor binding motif (RBM) of spikes in complex with ACE2. Mass spectra of the representative peptides of the RBM (listed in Supplementary Fig. 42) deconvoluted with bimodal or unimodal fittings with HX-Express 2. a) Mass spectra of the peptides of Wuhan, G614 and delta spikes. b) Mass spectra of the peptides of alpha, beta and omicron spikes. The horizontal orange lines indicate the distribution width at 50% BPI; the red dots mark the envelope peaks; the vertical green lines indicate the centroid mass; the dark blue lines fit the unimodal envelope distributions deconvoluted with binomial fitting; the green lines fit the low-mass envelope distributions deconvoluted with bimodal fitting; the light blue lines fit the high-mass envelope distributions deconvoluted with bimodal fitting.

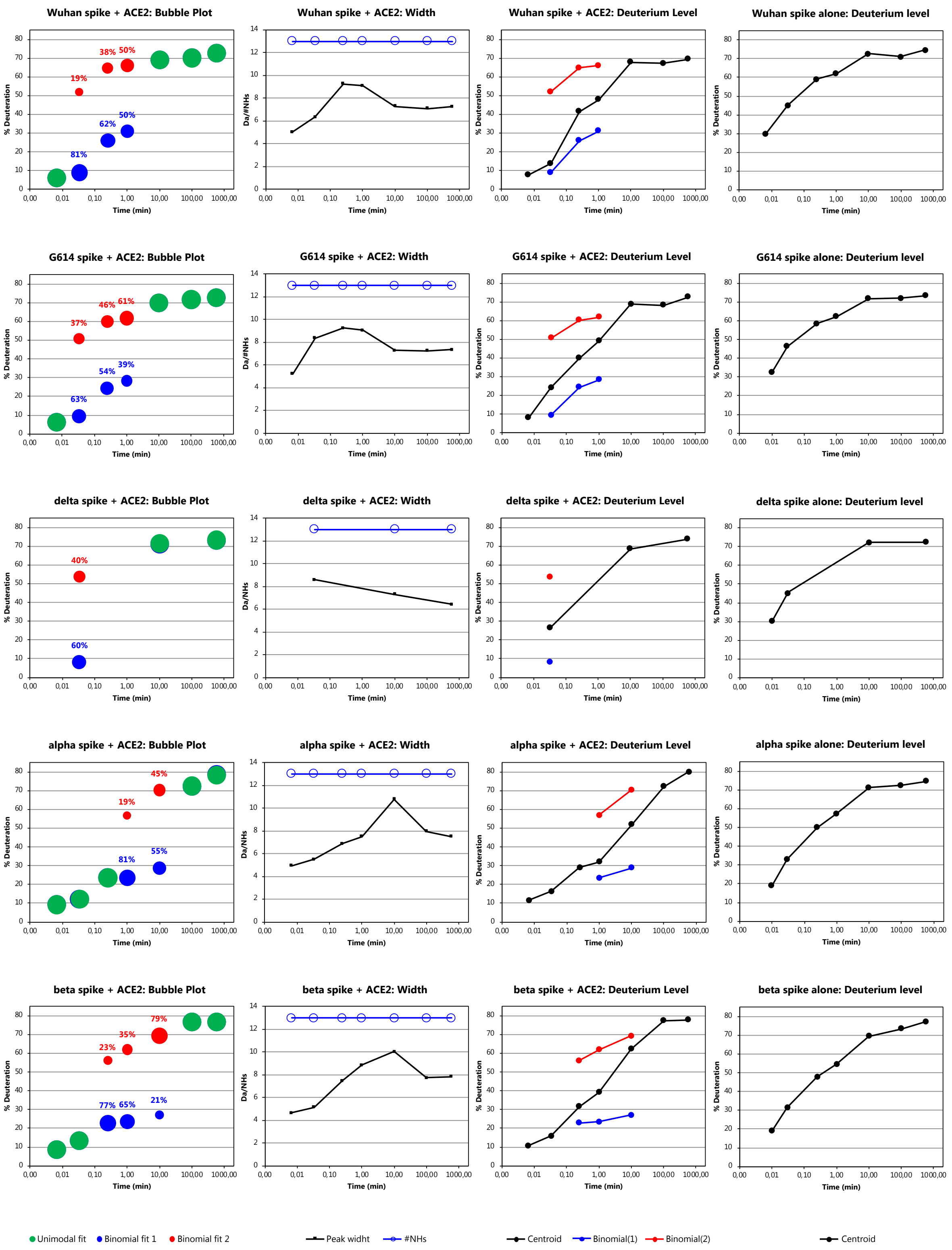
Wuhan spike + ACE2: Bubble Plot
Wuhan spike + ACE2: Width
Wuhan spike + ACE2: Deuterium Level
Wuhan spike alone: Deuterium level
G614 spike + ACE2: Bubble Plot
G614 spike + ACE2: Width
G614 spike + ACE2: Deuterium Level
G614 spike alone: Deuterium level
delta spike + ACE2: Bubble Plot
delta spike + ACE2: Width
delta spike + ACE2: Deuterium Level
delta spike alone: Deuterium level
alpha spike + ACE2: Bubble Plot
alpha spike + ACE2: Width
alpha spike + ACE2: Deuterium Level
alpha spike alone: Deuterium level
beta spike + ACE2: Bubble Plot
beta spike + ACE2: Width
beta spike + ACE2: Deuterium Level
beta spike alone: Deuterium level
% Deuteration
Da/#NHs
Time (min)
Unimodal fit
Binomial fit 1
Binomial fit 2
Peak widht
#NHs
Centroid
Binomial(1)
Binomial(2)
Centroid

Supplementary Fig. 44. Analysis of the bimodal HDX profiles in the receptor binding motif (RBM) of spikes in complex with ACE2. From top to bottom: peptide YGFQPTNGVGYQPYRV (495-510) of Wuhan spike; peptide YGFQPTNGVGYQPYRV (495-510) of G614 spike; peptide YGFQPTNGVGYQPYRV (493-509) of delta spike; peptide YGFQPTYGVGYQPYRV (492-507) of alpha spike; peptide YGFQPTYGVGYQPYRV (492-507) of beta spike. From left to right: bubble plots approximating the level of deuteration and the relative intensity of the low- and high-mass envelope (binomial fit 1 and binomial fit 2, respectively) for bimodal isotopic distributions and the level of deuteration of the unimodal envelope (unimodal fit), set at 100% intensity, in the ACE2 bound state; peak width of the isotopic distribution (calculated at 20% of Baseline Peak Intensity - BPI) and number of exchangble amides (#NHs), in the bound state; deuterium level of the low-mass envelopes (binomial 1), high-mass envelopes (binomial 2) and unimodal distributions (centroid) in ACE2-bound spike; deuterium level of spikes in apo state (unimodal distributions – centroid). Spectral analysis was conducted with HX-Express2[2] and graphs were authomatically generated upon fitting the isotopic envelopes shown in Supplementary Fig. 45. Bimodal fit was applied to peptide spectra at time points showing clear features of bimodality and/or enhanced peak width. These data are indicative and semi-quantitative and need to be regarded as trends. Note that the high-mass population does not manifest at early time points, thus does not follow the HDX of the unbound state.

a

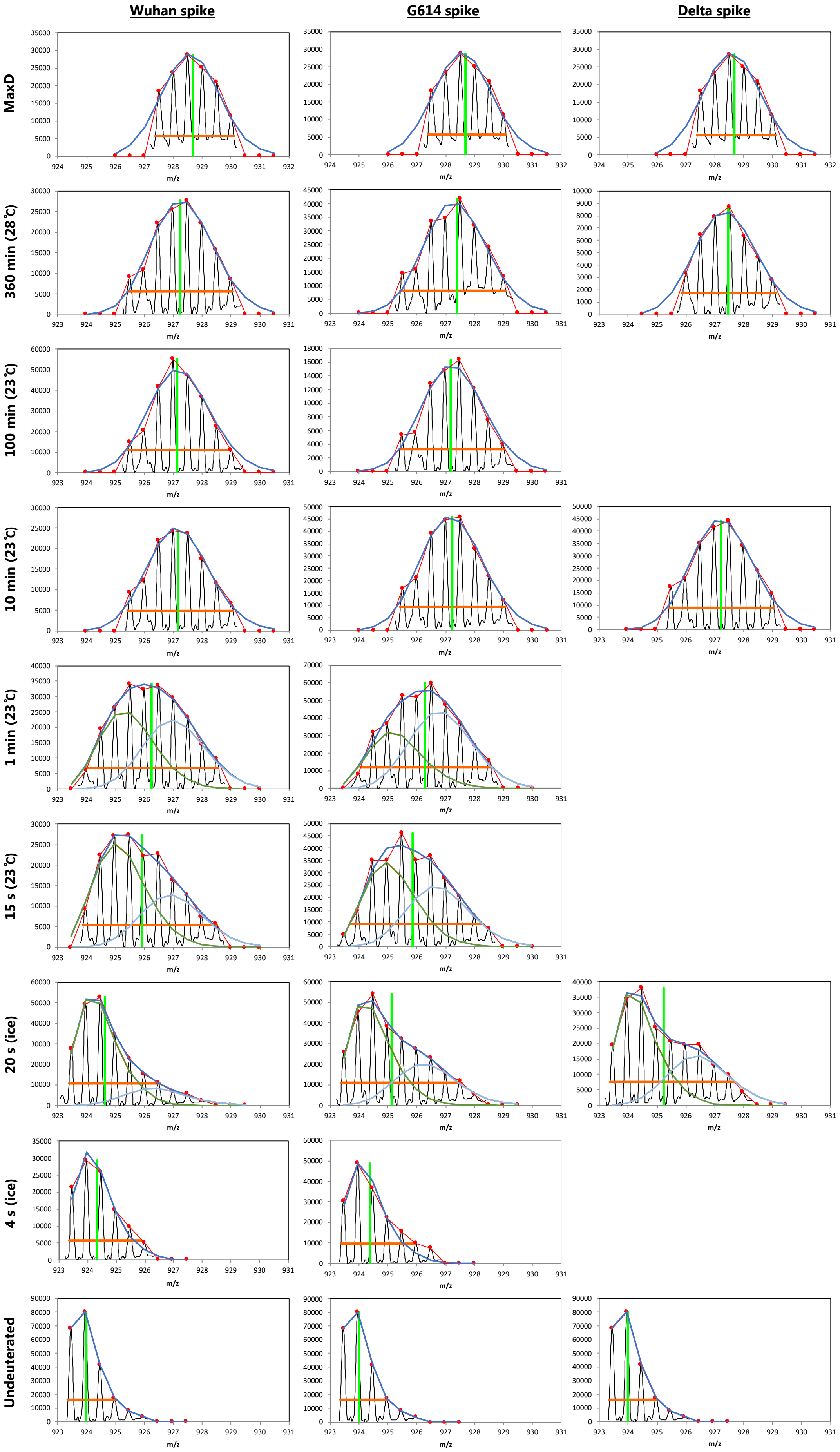
Wuhan spike
G614 spike
Delta spike
MaxD
360 min (28 ˚C)
100 min (23 ˚C)
10 min (23 ˚C)
1 min (23 ˚C)
15 s (23 ˚C)
20 s (ice)
4 s (ice)
Undeuterated
m/z

b

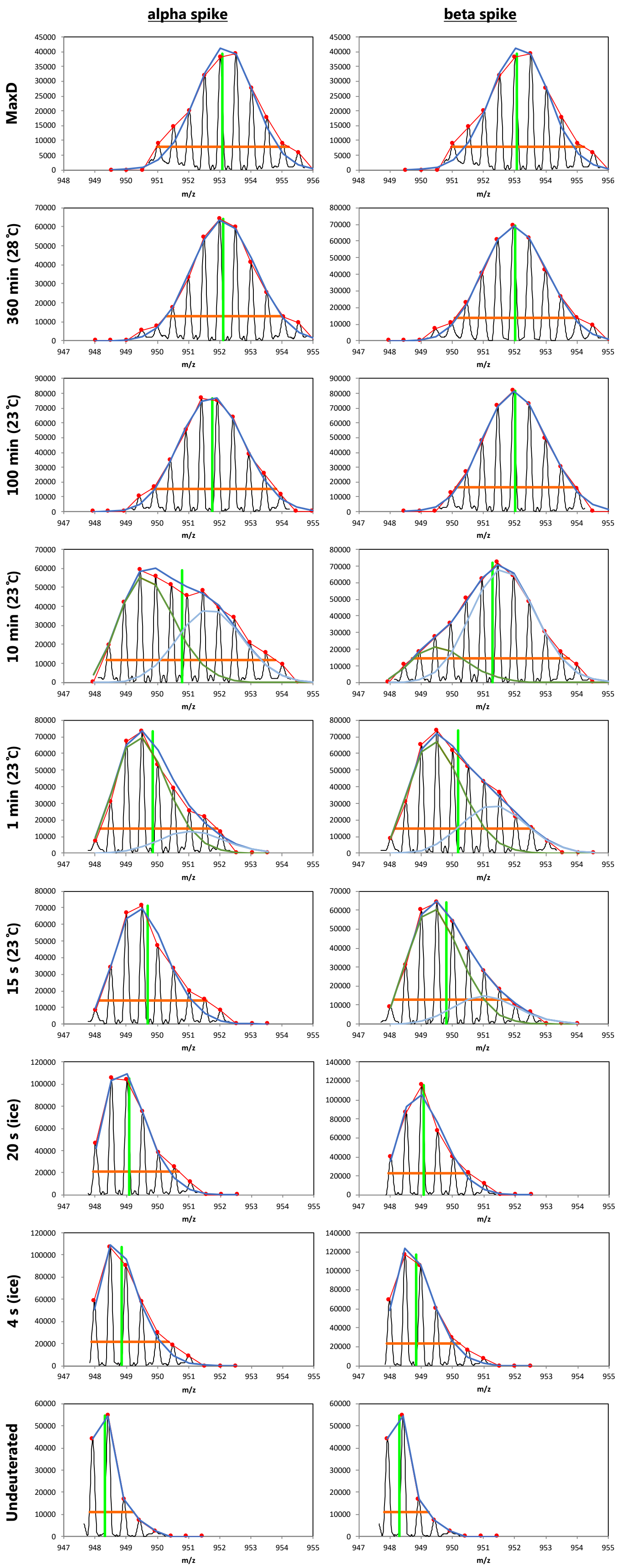
alpha spike
beta spike
MaxD
360 min (28 °C)
100 min (23 °C)
10 min (23 °C)
1 min (23 °C)
15 s (23 °C)
20 s (ice)
4 s (ice)
Undeuterated
m/z

Supplementary Fig. 45. Bimodal HDX profiles in the receptor binding motif (RBM) of spikes in complex with ACE2. Mass spectra of the representative peptides of the RBM (listed in Supplementary Fig. 44) deconvoluted with bimodal or unimodal fittings with HX-Express 2. a) Mass spectra of peptide of Wuhan, G614 and delta spikes. b) Mass spectra of peptide of alpha, beta and omicron spikes. The horizontal orange lines indicate the distribution width at 20% BPI; the red dots mark the envelope peaks; the vertical green lines indicate the centroid mass; the dark blue lines fit the unimodal envelope distributions deconvoluted with binomial fitting; the green lines fit the low-mass envelope distributions deconvoluted with bimodal fitting; the light blue lines fit the high-mass envelope distributions deconvoluted with bimodal fitting.

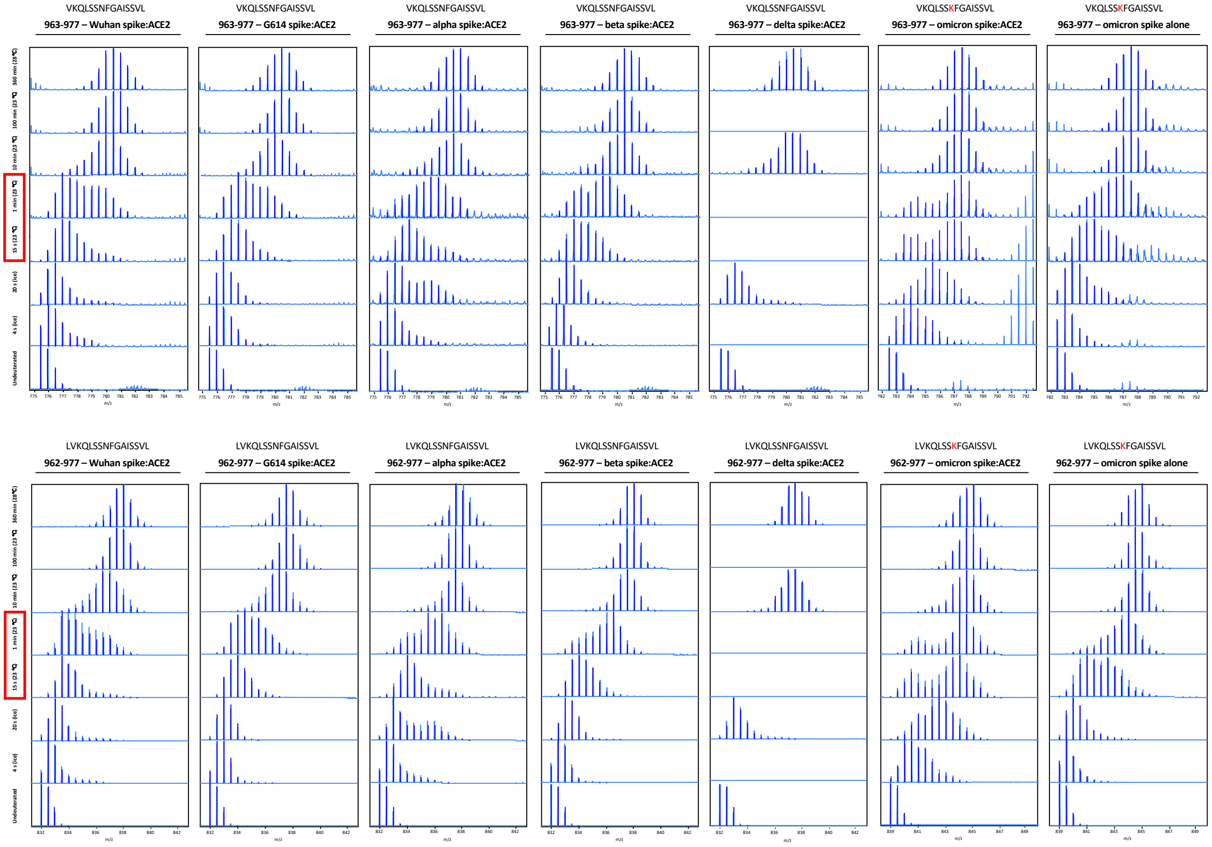

Supplementary Fig. 46. HDX bimodality in the HR1 of spikes in complex with ACE2 and omicron spike alone. Stacked spectral plots of peptides 962-977 and 963-977 illustrate the evolution of the bimodal isotopic envelopes over the time points studied. Particularly, time points 15 s and 1 min (23 °C) show clear separation between the low- and high-mass envelopes in every spike trimer, with the relative intensity and centroids of the two envelopes reporting on the degree of destabilization exerted by the ACE2 binding on the HR1. The isotopic envelopes in omicron spike alone manifest evident peak broadening, suggesting that the its HR1 follows a bimodal HDX behaviour even in the absence of ACE2.

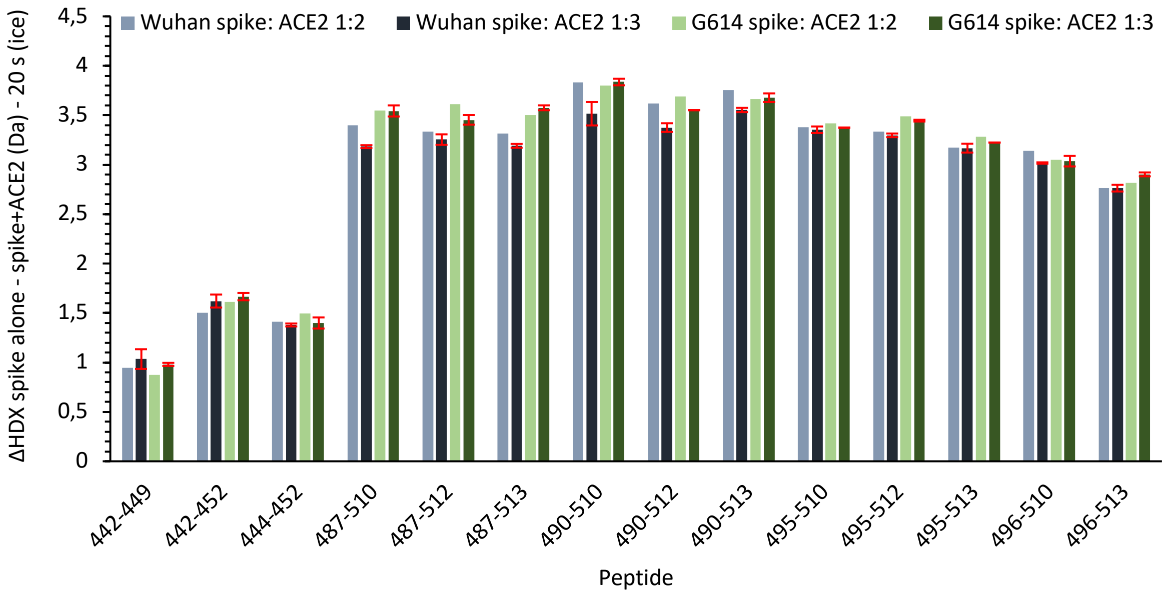

Supplementary Fig. 47. Histogram illustrating the magnitude of decreased HDX (ΔHDX) manifesting in selected peptides of the RBM when Wuhan and G614 spikes are incubated at 1:2 and 1:3 ratios with ACE2 (ratio 1:2 – single measurement; ratio 1:3 – duplicate measurement, presented as mean values +/- SD). No significant differences in ΔHDX are observed between the two incubation ratios and for both spike trimers. Source data are provided as a Source Data file.

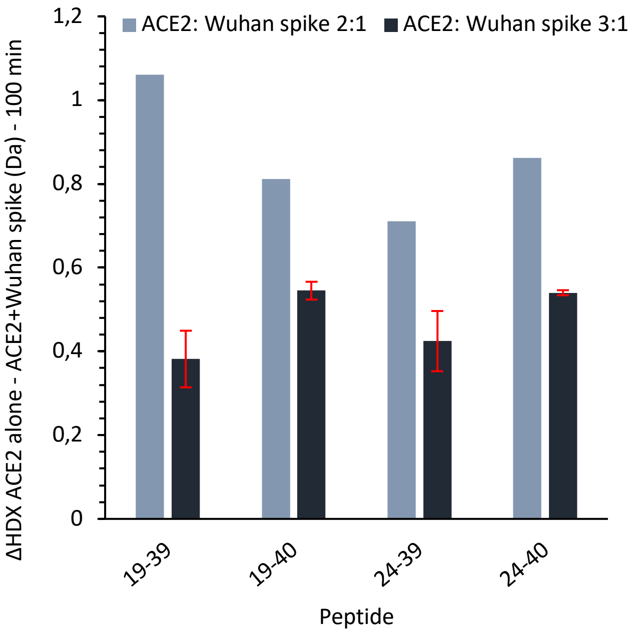

Supplementary Fig. 48. Histogram illustrating the magnitude of decreased HDX (ΔHDX) manifesting in selected peptides of the ACE2 binding site when ACE2 is incubated at 2:1 and 3:1 ratios with Wuhan spike (ratio 2:1 – single measurement; ratio 3:1 – duplicate measurement, presented as mean values +/- SD). A minor decrease in HDX is observed at ratio 3:1. Source data are provided as a Source Data file.

Supplementary tables 1-19

HDX summary tables of individual datasets, as per community-based recommendations[3]

Data Set	G614 spike	Wuhan spike
HDX reaction details	phosphate buffer saline (PBS), pD_{read} = 7.35, Deuterium fraction 84%, ratio 1:1	
HDX time course	4s (ice), 20 s (ice), 15 s (23 °C), 1 min (23 °C), 10 min (23 °C), 100 min (23 °C), 6 h (28 °C)	
HDX control samples	Maximally labeled control	
Back-exchange (mean / IQR)	25.32% / 10.49%	
# of Peptides / Average peptide lenght	308 / 11.92	
Sequence coverage	82%	
Replicates (technical)	duplicates - 4 s (ice); triplicates - 20 s (ice) and 10 min (23 °C)	
Repeatability (average standard deviation)	0,060533	0,055716
Significant differences in HDX (delta HDX > X D)	0.45 D (98% CI)	

Data Set	G614 spike	Alpha spike
HDX reaction details	phosphate buffer saline (PBS), pD_{read} = 7.35, Deuterium fraction 84%, ratio 1:1	
HDX time course	4s (ice), 20 s (ice), 15 s (23 °C), 1 min (23 °C), 10 min (23 °C), 100 min (23 °C), 6 h (28 °C)	
HDX control samples	Maximally labeled control	
Back-exchange (mean / IQR)	25.02% / 9.83%	
# of Peptides / Average peptide length	260 / 11.39	
Sequence coverage	78%	
Replicates (technical)	duplicates - 4 s (ice); triplicates - 20 s (ice) and 10 min (23 °C)	
Repeatability (average standard deviation)	0,061379	0,052542
Significant differences in HDX (delta HDX > X D)	0.46 D (98% CI)	

Data Set	G614 spike	Beta spike
HDX reaction details	phosphate buffer saline (PBS), pD_{read} = 7.35, Deuterium fraction 84%, ratio 1:1	
HDX time course	4s (ice), 20 s (ice), 15 s (23 °C), 1 min (23 °C), 10 min (23 °C), 100 min (23 °C), 6 h (28 °C)	
HDX control samples	Maximally labeled control	
Back-exchange (mean / IQR)	25.02% / 9.83%	
# of Peptides / Average peptide length	274 / 11.49	
Sequence coverage	80%	
Replicates (technical)	duplicates - 4 s (ice); triplicates - 20 s (ice) and 10 min (23 °C)	
Repeatability (average standard deviation)	0,061379	0,045600
Significant differences in HDX (delta HDX > X D)	0.44 D (98% CI)	

Data Set	G614 spike	Delta spike
HDX reaction details	phosphate buffer saline (PBS), pD_{read} = 7.35, Deuterium fraction 84%, ratio 1:1	
HDX time course	4s (ice), 20 s (ice), 15 s (23 °C)*, 1 min (23 °C)*, 10 min (23 °C), 100 min (23 °C)*, 6 h (28 °C); *absent in Delta spike	
HDX control samples	Maximally labeled control	
Back-exchange (mean / IQR)	25.02% / 9.83%	
# of Peptides / Average peptide length	288 / 11.92	
Sequence coverage	79%	
Replicates (technical)	duplicates - 4 s (ice); triplicates - 20 s (ice) and 10 min (23 °C)	duplicates - all time points
Repeatability (average standard deviation)	0,061379	0,043115
Significant differences in HDX (delta HDX > X D)	0.46 D (98% CI)	

Data Set	G614 spike	Omicron spike
HDX reaction details	phosphate buffer saline (PBS), pD_{read} = 7.35, Deuterium fraction 84%, ratio 1:1	
HDX time course	4s (ice), 20 s (ice), 15 s (23 °C), 1 min (23 °C), 10 min (23 °C), 100 min (23 °C), 6 h (28 °C)	
HDX control samples	Maximally labeled control	
Back-exchange (mean / IQR)	25.46% / 9.54%	
# of Peptides / Average peptide length	269 / 12.16	
Sequence coverage	75%	
Replicates (technical)	duplicates - 4 s (ice); triplicates - 20 s (ice) and 10 min (23 °C)	duplicates - 4 s (ice) and 20 s (ice); triplicates - 10 min (23 °C)
Repeatability (average standard deviation)	0,06184	0,045532
Significant differences in HDX (delta HDX > X D)	0.47 D (98% CI)	

Data Set	G614 spike	G614 spike + ACE2
HDX reaction details	phosphate buffer saline (PBS), pD_{read} = 7.35, Deuterium fraction 84%, ratio 1:2 spike:ACE2	
HDX time course	4s (ice), 20 s (ice), 15 s (23 °C), 1 min (23 °C), 10 min (23 °C), 100 min (23 °C), 6 h (28 °C)	
HDX control samples	Maximally labeled control	
Back-exchange (mean / IQR)	25.32% / 10.49%	
# of Peptides / Average peptide length	297 / 11.88	
Sequence coverage	80%	
Replicates (technical)	duplicates - 4 s (ice); triplicates - 20 s (ice) and 10 min (23 °C)	
Repeatability (average standard deviation)	0,060533	0,055640
Significant differences in HDX (delta HDX > X D)	0.44 D (98% CI)	

Data Set	Wuhan spike	Wuhan spike + ACE2
HDX reaction details	phosphate buffer saline (PBS), pD_{read} = 7.35, Deuterium fraction 84%, ratio 1:2 spike:ACE2	
HDX time course	4s (ice), 20 s (ice), 15 s (23 °C), 1 min (23 °C), 10 min (23 °C), 100 min (23 °C), 6 h (28 °C)	
HDX control samples	Maximally labeled control	
Back-exchange (mean / IQR)	25.32% / 10.49%	
# of Peptides / Average peptide length	297 / 11.88	
Sequence coverage	80%	
Replicates (technical)	duplicates - 4 s (ice); triplicates - 20 s (ice) and 10 min (23 °C)	
Repeatability (average standard deviation)	0,055716	0,066771
Significant differences in HDX (delta HDX > X D)	0.46 D (98% CI)	

Data Set	Alpha spike	Alpha spike + ACE2
HDX reaction details	phosphate buffer saline (PBS), pD_{read} = 7.35, Deuterium fraction 84%, ratio 1:2 spike:ACE2	
HDX time course	4s (ice), 20 s (ice), 15 s (23 °C), 1 min (23 °C), 10 min (23 °C), 100 min (23 °C), 6 h (28 °C)	
HDX control samples	Maximally labeled control	
Back-exchange (mean / IQR)	25.02% / 9.83%	
# of Peptides / Average peptide length	230 / 11.17	
Sequence coverage	73%	
Replicates (technical)	duplicates - 4 s (ice); triplicates - 20 s (ice) and 10 min (23 °C)	
Repeatability (average standard deviation)	0,052542	0,049007
Significant differences in HDX (delta HDX > X D)	0.39 D (98% CI)	

Data Set	Beta spike	Beta spike + ACE2
HDX reaction details	phosphate buffer saline (PBS), pD_{read} = 7.35, Deuterium fraction 84%, ratio 1:2 spike:ACE2	
HDX time course	4s (ice), 20 s (ice), 15 s (23 °C), 1 min (23 °C), 10 min (23 °C), 100 min (23 °C), 6 h (28 °C)	
HDX control samples	Maximally labeled control	
Back-exchange (mean / IQR)	25.02% / 9.83%	
# of Peptides / Average peptide length	246 / 11.35	
Sequence coverage	76%	
Replicates (technical)	duplicates - 4 s (ice); triplicates - 20 s (ice) and 10 min (23 °C)	
Repeatability (average standard deviation)	0,045600	0,044029
Significant differences in HDX (delta HDX > X D)	0.32 D (98% CI)	

Data Set	Delta spike	Delta spike + ACE2
HDX reaction details	phosphate buffer saline (PBS), pD_{read} = 7.35, Deuterium fraction 84%, ratio 1:2 spike:ACE2	
HDX time course	4s (ice)*, 20 s (ice), 10 min (23 °C), 6 h (28 °C; *absent in Delta spike + ACE2	
HDX control samples	Maximally labeled control	
Back-exchange (mean / IQR)	25.02% / 9.83%	
# of Peptides / Average peptide length	255 / 11.79	
Sequence coverage	78%	
Replicates (technical)	duplicates - all time points	triplicates - 20 s (ice)
Repeatability (average standard deviation)	0,043115	0,061032
Significant differences in HDX (delta HDX > X D)	0.44 D (98% CI)	

Data Set	Omicron spike	Omicron spike + ACE2
HDX reaction details	phosphate buffer saline (PBS), pD_{read} = 7.35, Deuterium fraction 84%, ratio 1:2 spike:ACE2	
HDX time course	4s (ice), 20 s (ice), 15 s (23 °C), 1 min (23 °C), 10 min (23 °C), 100 min (23 °C), 6 h (28 °C)	
HDX control samples	Maximally labeled control	
Back-exchange (mean / IQR)	25.46% / 9.54%	
# of Peptides / Average peptide length	217 / 11.53	
Sequence coverage	72%	
Replicates (technical)	duplicates - 4 s (ice) and 20 s (ice); triplicates - 10 min (23 °C)	
Repeatability (average standard deviation)	0,045532	0,047723
Significant differences in HDX (delta HDX > X D)	0.36 D (98% CI)	

Data Set	ancestral RBD	ancestral RBD + ACE2
HDX reaction details	phosphate buffer saline (PBS), pD_{read} = 7.35, Deuterium fraction 84%, ratio 3:2 RBD:ACE2	
HDX time course	20 s (ice), 10 min (23 °C), 6 h (28 °C)	
HDX control samples	-	
Back-exchange (mean / IQR)	-	
# of Peptides / Average peptide length	51 / 14.24	
Sequence coverage	82%	
Replicates (technical)	triplicates - 20 s (ice) and 10 min (23 °C); duplicates - 6 h (28 °C)	
Repeatability (average standard deviation)	0,048868	0,059668
Significant differences in HDX (delta HDX > X D)	0.44 D (98% CI)	

Data Set	ACE2	ACE2 + G614 spike
HDX reaction details	phosphate buffer saline (PBS), pD_{read} = 7.35, Deuterium fraction 84%, ratio 1:2 spike:ACE2	
HDX time course	4s (ice), 20 s (ice), 15 s (23 °C), 1 min (23 °C), 10 min (23 °C), 100 min (23 °C), 6 h (28 °C)	
HDX control samples	Maximally labeled control	
Back-exchange (mean / IQR)	31.47% / 12.50%	
# of Peptides / Average peptide length	167 / 10.83	
Sequence coverage	81%	
Replicates (technical)	duplicates - 4 s (ice); triplicates - 20 s (ice) and 10 min (23 °C)	
Repeatability (average standard deviation)	0,040089	0,044476
Significant differences in HDX (delta HDX > X D)	0.31 D (98%) / 0.44 D (99% CI)	

Data Set	ACE2	ACE2 + Wuhan spike
HDX reaction details	phosphate buffer saline (PBS), pD_{read} = 7.35, Deuterium fraction 84%, ratio 1:2 spike:ACE2	
HDX time course	4s (ice), 20 s (ice), 15 s (23 °C), 1 min (23 °C), 10 min (23 °C), 100 min (23 °C), 6 h (28 °C)	
HDX control samples	Maximally labeled control	
Back-exchange (mean / IQR)	31.47% / 12.50%	
# of Peptides / Average peptide length	167 / 10.83	
Sequence coverage	81%	
Replicates (technical)	duplicates - 4 s (ice); triplicates - 20 s (ice) and 10 min (23 °C)	
Repeatability (average standard deviation)	0,040089	0,048302
Significant differences in HDX (delta HDX > X D)	0.31 D (98%) / 0.44 D (99% CI)	

Data Set	ACE2	ACE2 + Alpha spike
HDX reaction details	phosphate buffer saline (PBS), pD_{read} = 7.35, Deuterium fraction 84%, ratio 1:2 spike:ACE2	
HDX time course	4s (ice), 20 s (ice), 15 s (23 °C), 1 min (23 °C), 10 min (23 °C), 100 min (23 °C), 6 h (28 °C)	
HDX control samples	Maximally labeled control	
Back-exchange (mean / IQR)	30.47% / 12.55%	
# of Peptides / Average peptide length	167 / 10.83	
Sequence coverage	81%	
Replicates (technical)	duplicates - 4 s (ice); triplicates - 20 s (ice) and 10 min (23 °C)	
Repeatability (average standard deviation)	0,035612	0,041715
Significant differences in HDX (delta HDX > X D)	0.28 D (98%) / 0.39 D (99% CI)	

Data Set	ACE2	ACE2 + Beta spike
HDX reaction details	phosphate buffer saline (PBS), pD_{read} = 7.35, Deuterium fraction 84%, ratio 1:2 spike:ACE2	
HDX time course	4s (ice), 20 s (ice), 15 s (23 °C), 1 min (23 °C), 10 min (23 °C), 100 min (23 °C), 6 h (28 °C)	
HDX control samples	Maximally labeled control	
Back-exchange (mean / IQR)	30.47% / 12.55%	
# of Peptides / Average peptide length	167 / 10.83	
Sequence coverage	81%	
Replicates (technical)	duplicates - 4 s (ice); triplicates - 20 s (ice) and 10 min (23 °C)	
Repeatability (average standard deviation)	0,035612	0,043306
Significant differences in HDX (delta HDX > X D)	0.28 D (98%) / 0.40 D (99% CI)	

Data Set	ACE2	ACE2 + Delta spike
HDX reaction details	phosphate buffer saline (PBS), pD_{read} = 7.35, Deuterium fraction 84%, ratio 1:2 spike:ACE2	
HDX time course	4s (ice)*, 20 s (ice), 15 s (23 °C)*, 1 min (23 °C)*, 10 min (23 °C), 100 min (23 °C)*, 6 h (28 °C); absent in Delta spike + ACE2	
HDX control samples	Maximally labeled control	
Back-exchange (mean / IQR)	30.47% / 12.55%	
# of Peptides / Average peptide length	167 / 10.83	
Sequence coverage	81%	
Replicates (technical)	duplicates - 4 s (ice); triplicates - 20 s (ice) and 10 min (23 °C)	triplicates - 20 s (ice)
Repeatability (average standard deviation)	0,035612	0,043306
Significant differences in HDX (delta HDX > X D)	0.27 D (98%) / 0.39 D (99% CI)	

Data Set	ACE2	ACE2 + Omicron spike
HDX reaction details	phosphate buffer saline (PBS), pD_{read} = 7.35, Deuterium fraction 84%, ratio 1:2 spike:ACE2	
HDX time course	4s (ice), 20 s (ice), 15 s (23 °C), 1 min (23 °C), 10 min (23 °C), 100 min (23 °C), 6 h (28 °C)	
HDX control samples	Maximally labeled control	
Back-exchange (mean / IQR)	33.16% / 11.29%	
# of Peptides / Average peptide length	167 / 10.83	
Sequence coverage	81%	
Replicates (technical)	duplicates - 4 s (ice); triplicates - 20 s (ice) and 10 min (23 °C)	duplicates - 4 s (ice) and 20 s (ice); triplicates - 10 min (23 °C)
Repeatability (average standard deviation)	0,036086	0,036658
Significant differences in HDX (delta HDX > X D)	0.27 D (98%) / 0.39 D (99% CI)	

Data Set	ACE2	ACE2 + ancestral RBD
HDX reaction details	phosphate buffer saline (PBS), pD_{read} = 7.35, Deuterium fraction 84%, ratio 3:2 RBD:ACE2	
HDX time course	20 s (ice), 10 min (23 °C), 6 h (28 °C)	
HDX control samples	-	
Back-exchange (mean / IQR)	-	
# of Peptides / Average peptide length	166 / 10.85	
Sequence coverage	81%	
Replicates (technical)	triplicates - 20 s (ice) and 10 min (23 °C); duplicates - 6 h (28 °C)	
Repeatability (average standard deviation)	0,045865	0,040228
Significant differences in HDX (delta HDX > X D)	0.31 D (98%) / 0.44 D (99% CI)	

Supplementary references

1. Kavan, D. & Man, P. MSTools - Web based application for visualization and presentation of HXMS data. *Int. J. Mass Spectrom.* **302**, 53–58 (2011).
2. Guttman, M., Weis, D. D., Engen, J. R. & Lee, K. K. Analysis of Overlapped and Noisy Hydrogen/Deuterium Exchange Mass Spectra. *J. Am. Soc. Mass Spectrom.* **24**, 1906–1912 (2013).
3. Masson, G. R. *et al.* Recommendations for performing, interpreting and reporting hydrogen deuterium exchange mass spectrometry (HDX-MS) experiments. *Nat. Methods* **16**, 595–602 (2019).
