# Supplementary material for: Structural dynamics in the evolution of SARS-CoV-2 spike glycoprotein
Source: Nat Commun. 2023 Mar 14;14:1421. doi: 10.1038/s41467-023-36745-0 (PMC10013288; doi:10.1038/s41467-023-36745-0)
Supplement: Supplementary file 3 — Description of Additional Supplementary Files [file 41467_2023_36745_MOESM3_ESM.pdf]

Description of Additional Supplementary Information File

Title: Supplementary Data

Description: Analysis of the impact of amino acid changes on the kch and HDX of spike variants.
