# Supplementary material for: Structural dynamics in the evolution of SARS-CoV-2 spike glycoprotein
Source: Nat Commun. 2023 Mar 14;14:1421. doi: 10.1038/s41467-023-36745-0 (PMC10013288; doi:10.1038/s41467-023-36745-0)
Supplement: Supplementary file 5 — Reporting Summary [file 41467_2023_36745_MOESM5_ESM.pdf]

nature portfolio

Corresponding author(s): Valeria Calvares, Antoni Wrobel, Argyris Politis

Last updated by author(s): Feb 3, 2023

Reporting Summary

Nature Portfolio wishes to improve the reproducibility of the work that we publish. This form provides structure for consistency and transparency in reporting. For further information on Nature Portfolio policies, see our Editorial Policies and the Editorial Policy Checklist.

Statistics

For all statistical analyses, confirm that the following items are present in the figure legend, table legend, main text, or Methods section.

n/a	Confirmed	
☐	☒	The exact sample size (*n*) for each experimental group/condition, given as a discrete number and unit of measurement
☐	☒	A statement on whether measurements were taken from distinct samples or whether the same sample was measured repeatedly
☐	☒	The statistical test(s) used AND whether they are one- or two-sided<br>*Only common tests should be described solely by name; describe more complex techniques in the Methods section.*
☒	☐	A description of all covariates tested
☐	☒	A description of any assumptions or corrections, such as tests of normality and adjustment for multiple comparisons
☐	☒	A full description of the statistical parameters including central tendency (e.g. means) or other basic estimates (e.g. regression coefficient) AND variation (e.g. standard deviation) or associated estimates of uncertainty (e.g. confidence intervals)
☒	☐	For null hypothesis testing, the test statistic (e.g. *F*, *t*, *r*) with confidence intervals, effect sizes, degrees of freedom and *P* value noted<br>*Give P values as exact values whenever suitable.*
☒	☐	For Bayesian analysis, information on the choice of priors and Markov chain Monte Carlo settings
☒	☐	For hierarchical and complex designs, identification of the appropriate level for tests and full reporting of outcomes
☒	☐	Estimates of effect sizes (e.g. Cohen's *d*, Pearson's *r*), indicating how they were calculated

Our web collection on statistics for biologists contains articles on many of the points above.

Software and code

Policy information about availability of computer code

Data collection	MassLynx (v. 4.1, Waters) was used for controlling the Synapt G2-Si mass spectrometer and for the acquisition of MS and MS/MS data.
Data analysis	ProteinLynx Global Server (v. 3.0, Waters) was used for processing MS/MS data. DynamX (v. 3.0, Waters) was used for processing HDX-MS data. HDX bimodal envelope distributions were analyzed by HX-Express (version 2). Statistical analysis has been performed with Deuteros (version 1).

For manuscripts utilizing custom algorithms or software that are central to the research but not yet described in published literature, software must be made available to editors and reviewers. We strongly encourage code deposition in a community repository (e.g. GitHub). See the Nature Portfolio guidelines for submitting code & software for further information.

Data

Policy information about availability of data

All manuscripts must include a data availability statement. This statement should provide the following information, where applicable:

- Accession codes, unique identifiers, or web links for publicly available datasets
- A description of any restrictions on data availability
- For clinical datasets or third party data, please ensure that the statement adheres to our policy

According to the community-based recommendations, to allow access to the HDX data of this study, the HDX summary tables are included as supporting tables 1-19, the HDX data tables are provided as source data with this paper and all deuterium uptake plots are deposited in the figshare repository at https://

figshare.com/s/f1db735795acd604e470. The HDX mass spectrometry data have been deposited to the ProteomeXchange Consortium via the PRIDE partner repository with the dataset identifiers PXD039760 and PXD039762.

Human research participants

Policy information about studies involving human research participants and Sex and Gender in Research.

Reporting on sex and gender	This study does not involve human research participants.
Population characteristics	This study does not involve human research participants.
Recruitment	This study does not involve human research participants.
Ethics oversight	This study does not involve human research participants.

Note that full information on the approval of the study protocol must also be provided in the manuscript.

Field-specific reporting

Please select the one below that is the best fit for your research. If you are not sure, read the appropriate sections before making your selection.

☒ Life sciences ☐ Behavioural & social sciences ☐ Ecological, evolutionary & environmental sciences

For a reference copy of the document with all sections, see nature.com/documents/nr-reporting-summary-flat.pdf

Life sciences study design

All studies must disclose on these points even when the disclosure is negative.

Sample size	No sample size calculation has been performed, as not relevant for this study. Guidelines on sample size have been established and provided by the HDX-MS community-based recommendations (Masson et al., Nat Methods, 2019). Following the HDX-MS community-based recommendations, at least two data points have been performed in triplicates for each HDX dataset (the requirement is at least one time point).
Data exclusions	Peptides were excluded if they were insufficiently fragmented, if their signal-to-noise ratio was not suitable for accurate HDX assessment and/or if the mass error was above 10 ppm. A list of included peptides can be found in supplementary figures and in source data.
Replication	Experiments were performed in technical replicates; at least two data points have been performed in triplicates for each HDX dataset. Parameters on replication (average SD) of each protein state in each HDX dataset are provided in the supplementary tables 1-19 and used to derive the threshold of significance. The replication was successful.
Randomization	Samples were allocated for injections in the LC-MS apparatus according the HDX labeling time (i.e. each labelling time point was analyzed to completion before starting a new time point), with the limitation of the available machine time. This allocation minimizes the confounding factor of difference in back-exchange between protein samples of the same labelling time. Samples were injected in a random order within the individual data points and states.
Blinding	Blinding was not relevant for this study. The identity of the proteins analyzed and samples injected needs to be known to correctly match the raw data and peptide search list.

Reporting for specific materials, systems and methods

We require information from authors about some types of materials, experimental systems and methods used in many studies. Here, indicate whether each material, system or method listed is relevant to your study. If you are not sure if a list item applies to your research, read the appropriate section before selecting a response.

Materials & experimental systems

n/a	Involved in the study	
☒	☐	Antibodies
☐	☒	Eukaryotic cell lines
☒	☐	Palaeontology and archaeology
☒	☐	Animals and other organisms
☒	☐	Clinical data
☒	☐	Dual use research of concern

Methods

n/a	Involved in the study	
☒	☐	ChIP-seq
☒	☐	Flow cytometry
☒	☐	MRI-based neuroimaging

Eukaryotic cell lines

Policy information about cell lines and Sex and Gender in Research

Cell line source(s)	expi293F cells were purchased from Thermo Scientific and used for protein expression
Authentication	Cell line used was not authenticated, although it came from a commercial source
Mycoplasma contamination	Cell line was not tested for mycoplasma contamination
Commonly misidentified lines (See ICLAC register)	No commonly misidentified cell lines were used in this study
